# PfbZIP85 Transcription Factor Mediates ω-3 Fatty Acid-Enriched Oil Biosynthesis by Down-Regulating *PfLPAT1B* Gene Expression in Plant Tissues

**DOI:** 10.3390/ijms25084375

**Published:** 2024-04-16

**Authors:** Xusheng Huang, Yali Zhou, Xianfei Shi, Jing Wen, Yan Sun, Shuwei Chen, Ting Hu, Runzhi Li, Jiping Wang, Xiaoyun Jia

**Affiliations:** 1 College of Agronomy/Institute of Molecular Agriculture & Bioenergy, Shanxi Agricultural University, Shanxi Engineering Research Center for Genetics and Metabolism of Specific Crops, Jinzhong 030801, China; hxss214809@163.com (X.H.); zhouylnd@163.com (Y.Z.); sxndwjp@sxau.edu.cn (J.W.); 2 College of Life Sciences, Shanxi Agricultural University, Jinzhong 030801, China

**Keywords:** perilla (*Perilla frutescens*), bZIP transcription factors, genome-wide identification, expression analysis, oil biosynthesis and regulation, lysophosphatidate acyltransferase (LPAT)

## Abstract

The basic leucine zipper (bZIP) transcription factor (TF) family is one of the biggest TF families identified so far in the plant kingdom, functioning in diverse biological processes including plant growth and development, signal transduction, and stress responses. For *Perilla frutescens*, a novel oilseed crop abundant in polyunsaturated fatty acids (PUFAs) (especially α-linolenic acid, ALA), the identification and biological functions of bZIP members remain limited. In this study, 101 PfbZIPs were identified in the perilla genome and classified into eleven distinct groups (Groups A, B, C, D, E, F, G, H, I, S, and UC) based on their phylogenetic relationships and gene structures. These *PfbZIP* genes were distributed unevenly across 18 chromosomes, with 83 pairs of them being segmental duplication genes. Moreover, 78 and 148 pairs of orthologous *bZIP* genes were detected between perilla and *Arabidopsis* or sesame, respectively. PfbZIP members belonging to the same subgroup exhibited highly conserved gene structures and functional domains, although significant differences were detected between groups. RNA-seq and RT-qPCR analysis revealed differential expressions of 101 *PfbZIP* genes during perilla seed development, with several *PfbZIPs* exhibiting significant correlations with the key oil-related genes. Y1H and GUS activity assays evidenced that *PfbZIP85* downregulated the expression of the *PfLPAT1B* gene by physical interaction with the promoter. *PfLPAT1B* encodes a lysophosphatidate acyltransferase (LPAT), one of the key enzymes for triacylglycerol (TAG) assembly. Heterogeneous expression of *PfbZIP85* significantly reduced the levels of TAG and UFAs (mainly C18:1 and C18:2) but enhanced C18:3 accumulation in both seeds and non-seed tissues in the transgenic tobacco lines. Furthermore, these transgenic tobacco plants showed no significantly adverse phenotype for other agronomic traits such as plant growth, thousand seed weight, and seed germination rate. Collectively, these findings offer valuable perspectives for understanding the functions of PfbZIPs in perilla, particularly in lipid metabolism, showing PfbZIP85 as a suitable target in plant genetic improvement for high-value vegetable oil production.

## 1. Introduction

*Perilla frutescens* (2*n* = 40), commonly called perilla, is one of the annual herbs belonging to the *Lamiaceae* family [1]. There are two different varieties, namely *P. frutescens* var. *frutescens* and *P. frutescens* var. *crisp*, respectively. *P. frutescens* var. *frutescens* serves as an oilseed crop used for vegetable oil production, while *P. frutescens* var. *crisp* is utilized as an herb for its fragrant and flavorful leaves [2,3]. Perilla is native to China and nowadays is extensively planted in various Asian countries such as Korea, Japan, and India [4,5,6]. As a traditional edible and medicinal herb in Asia, perilla is broadly used as raw materials for food, healthcare products, pharmaceuticals, cosmetics, and other industries production because of the multiple nutrients and bioactive substances identified from this plant [6,7,8]. More importantly, perilla seed oil serves as an abundant source of polyunsaturated fatty acids (PUFAs), especially ω-3 FA (α-linolenic acid, ALA, 18:3 Δ^9,12,15^) with a high content of 54~64% and ω-6 FA (linoleic acid, LA, 18:2 Δ^9,12^) with a content of about 14% [2,9]. ALA plays an important role in maintaining the bio-membrane fluidity of plants and serves as a precursor to signaling molecular substances (e.g., jasmonic acid, JA) derived from FA metabolism, which mediate plant growth, development, and stress responses [5,10]. ALA is also a unique substrate for the synthesis of EPA (eicosapentaenoic acid, C20:5 Δ^5,8,11,14,17^) and DHA (docosahexaenoic acid, C22:6 Δ^4,7,10,13,16,19^) [5]. These three PUFAs unable to be synthesized in the human body have critical health benefits for humans, including regulating body development, promoting brain nutrition, improving memory, enhancing blood circulation, reducing both blood pressure and lipids, and repressing senescence, with therapeutic benefits for neurological disorders as well as cardiovascular and cerebrovascular diseases [5,11]. To date, perilla seed oil has been identified as one of the highest ALA accumulators among vegetable oils and recognized as a high-quality resource for essential ALA in the human diet. Therefore, in-depth understanding of the biosynthesis and regulatory mechanisms of perilla seed oil is of significance for the genetic improvement of perilla oil production and oil quality to sustainably produce vegetable oils rich in ω-3 FAs.

Plant lipid biosynthesis is regulated by multiple regulatory pathways. Transcription factor (TF) is a crucial player involved in such complex regulatory networks, which can coordinate various biological processes by up- or down-regulating the expressions of the related genes, or TFs [12,13]. So far, many TFs have been tested as regulators in lipid/FA biosynthesis and other biological processes of plants. These TFs include LEC1 [14], LEC2, FUS3, ABI3 [15], WRI1 [16], MYB [17], WRKY [18], NAC [19], bZIP [20], and Dof [21]. Among them, the bZIP (basic leucine zipper) TF family with larger numbers of members was found to function diversely. The bZIP members have a highly conserved domain that consists of two typical structural elements: the basic region (BR) and the leucine zipper (LZ) dimerization motif [22,23]. Plant bZIP TFs preferentially bind to DNA sequences containing core elements of ACGT (for instance, the CACGTG (G-box), GACGTC (C-box), or TACGTA (A-box)) [22,24,25].

Currently, bZIP family members have been identified or predicted in various eukaryotic genomes, such as those of plants, animals, and yeasts [23,26,27,28]. For example, genome-wide identification of bZIP members was conducted in Arabidopsis [26], sorghum [23], rice [27], castor bean [25], maize [29], gymnosperms, and angiosperms [30], showing significant differences in the number of bZIP TF members among these plant species. For instance, 75, 89, 49, 160, 50, 125, 63, and 247 bZIP members were identified, respectively, in *Arabidopsis* [26], rice (*Oryza sativa*) [27], maize (*Zea mays*) [29], castor bean (*Ricinus communis*) [25], rapeseed (*Brassica napus*) [31], soybean (*Glycine max*) [32], sesame (*Sesamum indicum*) [33], and peanut (*Arachis duranensis*) [34] (Table S1). Moreover, bZIP TFs are detected to regulate diverse plant developmental and physiological processes [23,35]. Several bZIP TFs are found to play key roles in plant organogenesis and tissue differentiation [36,37,38], nitrogen/carbon cycling and energy metabolism [39,40,41], as well as the regulation of seed storage protein gene expression [42]. In addition, bZIP TFs are considered crucial regulators in modulating responses to diverse abiotic stresses, including drought, cold, and high salinity stresses in *Arabidopsis* [43], wheat [44], soybean [45,46], and rice [47,48]. A few bZIP TFs are also identified to mediate lipid metabolism in plants [49]. In *Arabidopsis*, AtbZIP67 can bind to the G-box-acting element located in the promoter region of the *FAD3* gene to increase *FAD3* expression, leading to an improvement in ω-3 PUFA content in the seeds [50]. Overexpression of soybean *GmbZIP123* in *Arabidopsis* resulted in an enhancement of seed lipid content [51], indicating that *GmbZIP123* can regulate lipid biosynthesis in seeds. The bZIP mutant *crbzip2* of *Chlamydomonas reinhardtii* CC-5325 was observed to decrease the accumulation of TAGs while increasing the accumulation of polar lipids, particularly digalactosyldiacylglycerol (DGDG), under nitrogen-deficient conditions [52]. The overexpression of the *NsbZIP1* gene generated an enhancement of neutral lipid content and a higher level of fatty acid methyl ester (FAME) compared to the wild-type *Nannochloropsis salina* algae CCMP1776 under nitrogen stress conditions. Furthermore, the overexpression of *NsbZIP1* led to an upregulation of genes involved in lipid metabolism, including *ACBP*, *KAS*, *LC-FACS*, and *LPAAT* [53].

Collectively, bZIP TFs are found to mediate multiple life processes, including signal transduction, metabolism, growth, and development, despite different bZIP members function diversely. However, limited information is obtained for bZIP TF family members and their biological roles in perilla. Based on the known knowledge that a few bZIP TFs functioned in the regulation of lipid metabolism in some oleaginous seeds, we hypothesized that some members of PfbZIP TFs may also mediate the biosynthesis and regulation of perilla seed oils enriched with ω-3 FAs. Therefore, this study was conducted for a systematic analysis of PfbZIP TFs in perilla, aiming to elucidate their functions, particularly the PfbZIP-mediated regulation internet responsible for the biosynthesis of seed oils enriched with ω-3 FAs. The genome-wide identification of the *PfbZIP* genes was performed using omics-based approaches, including chromosome localization, sequence characteristics, gene structure, conserved motifs, promoter cis-acting elements, phylogenetic relationships, gene duplication, and intergenomic synteny analysis. Moreover, the expression patterns of *PfbZIP* genes in various tissues of perilla were extensively examined using RNA-Seq and further validated by quantitative real-time RT-PCR (RT-qPCR) in perilla seeds at various developmental stages. Finally, yeast single hybridization (Y1H) and transient co-expression assays revealed that PfbZIP85 repressed the expression of *PfLPAT1B* (a key gene for TAG assembly) by physical interaction with the gene promoter. Overexpression of *PfbZIP85* resulted in a significant change in oil and main FA levels in both leaves and seeds of the transgenic tobacco plants, accompanied by alternations in expressions of the lipid-related genes, including the *DGAT3* gene crucial for TAG assembly and other enzyme genes. This study generates a new scientific foundation for further analysis of the biological roles of PfbZIP TFs, offering valuable genetic resources for molecular breeding to enhance oil production and quality in perilla and other oil crops.

## 2. Results

### 2.1. A Total of 101 PfbZIP Transcription Factors Were Identified from P. frutescens

A total of 101 PfbZIP proteins were characterized from the genome database of *P. frutescens* (https://www.ncbi.nlm.nih.gov/genbank; accessed on 9 March 2023) [54] by BLAST analysis. All PfbZIP TFs were renamed from PfbZIP1 to PfbZIP101. The basic physical and chemical properties of those PfbZIPs were predicted using bioinformatics, including their gene ID, physical positions of the gene on chromosomes, length of protein sequence (aa), relative molecular weight (MW), isoelectric point (pI), and grouping of all gene members (Table S2). The protein length ranged from 112 aa (PfbZIP46 and PfbZIP98) to 1269 aa (PfbZIP89), with an average of 342 aa. Their MWs ranged from 12.07 kDa (PfbZIP46) to 142.58 kDa (PfbZIP89), with an average of 37.88 kDa. The pIs of the PfbZIPs varied from 4.84 (PfbZIP14) to 10.88 (PfbZIP46), with an average of 7.10. Compared to the numbers of bZIP family members published in other plant species (Table S1), the PfbZIP family had larger members, suggesting that the PfbZIP family was extensively expanded in *P. frutescens* during evolution.

To ascertain the chromosomal localization of *PfbZIP* genes, all identified *PfbZIP* genes were displayed on the chromosomes of perillas using MapChart 2.32 [55]. A total of 101 *PfbZIP* genes were distributed differentially on 18 chromosomes of perilla (Figure S1) without the *PfbZIP* gene on chromosomes 11 and 18. For example, chromosomes 12, 1, and 16 contained 11 and 10 *PfbZIP* genes, respectively, while two *PfbZIPs* were mapped to chromosomes 5 and 17, respectively. On the other chromosomes, the number of *PfbZIP* genes varied from three to eight.

### 2.2. Phylogenetic Analysis of PfbZIP Proteins

**Figure 1:**
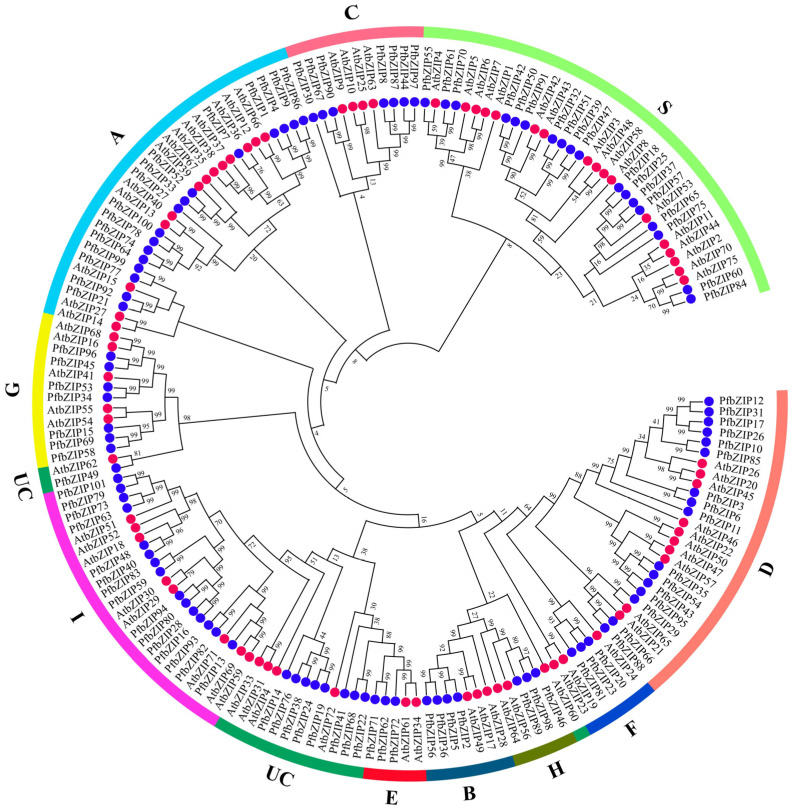
Phylogenetic tree of bZIP members from *P. frutescens* and *Arabidopsis*. The PfbZIP proteins (blue solid circle) and AtbZIP proteins (red solid circle) were classified into distinct groups in the phylogenetic tree. Different colors on the periphery indicate the distinct subgroups of bZIP proteins.

**Figure 2:**
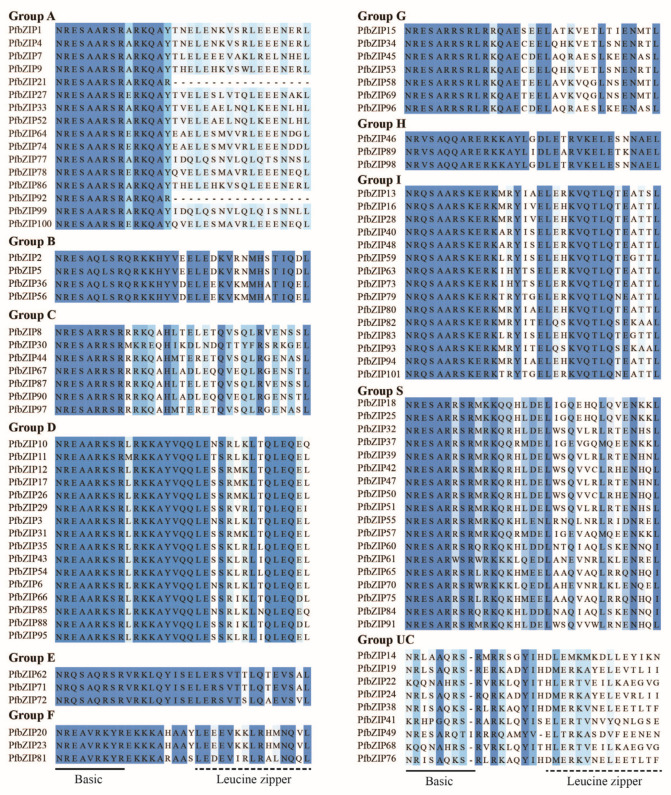
Multiple sequence alignments of the PfbZIP protein domains from *P. frutescens*. The basic region (BR) and leucine zipper (LZ) are shown by solid and dotted lines, respectively.

A phylogenetic tree was generated utilizing the neighbor-joining (N-J) method by MEGA 11.0.9 software, which included 75 AtbZIP proteins and 101 PfbZIP proteins (Figure 1). Based on the grouping of AtbZIP in *Arabidopsis*, PfbZIP members were divided into 11 distinct groups (Group A, B, C, D, E, F, G, H, I, and S, as well as an unclassified group UC), with 16, 4, 7, 16, 3, 3, 7, 3, 15, 18, and 9 PfbZIP proteins in each group, respectively. Furthermore, multiple sequence alignments of PfbZIP proteins with Jalview 2.11.2.7 software (Figure 2) demonstrated that most PfbZIP proteins had a basic region composed of N-x7-R/K at the N-terminal, followed by the conserved RK (or KK) and leucine zipper domains (L-x6-L-x6-L), which are typical features of bZIP proteins. Additionally, leucine (Leu) residues are substituted with valine (Val), isoleucine (Ile), or methionine (Met) in several PfbZIP proteins.

### 2.3. Segmental Duplication and Synteny Analysis of PfbZIP Genes

**Figure 3:**
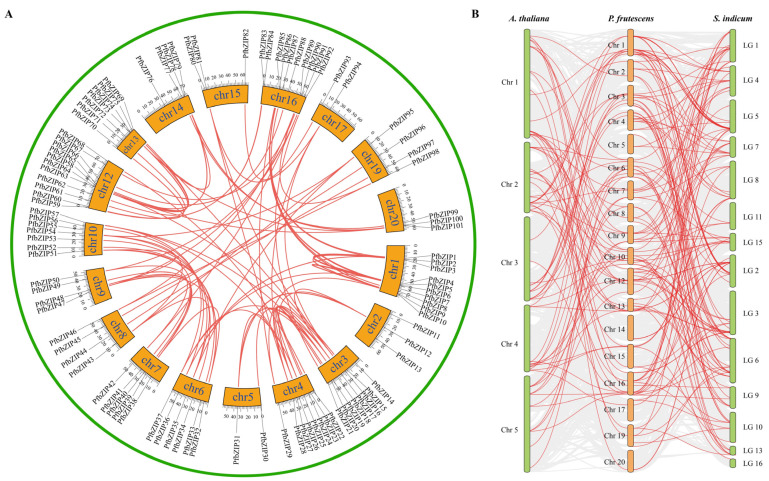
Synteny analysis of bZIP members between *P. frutescens* and the other two plant species. (**A**) Synteny examination of inter-chromosomal associations of *PfbZIP* genes. All *PfbZIP* gene pairs in the *P. frutescens* genome were indicated by red lines. (**B**) Synteny of *bZIP* members between *P. frutescens*, *A. thaliana*, and *S. indicum*. The synteny bZIP gene pairs between *P. frutescens*, *A. thaliana*, or *S. indicum* were emphasized using red lines. The collinear blocks of other genes between *P. frutescens* and *A. thaliana*, or *S. indicum*, were shown with gray lines.

It is well known that the expansion of a gene family is attributed to repetitive events occurring within the entire gene family or among certain members [12]. In comparison to other reported plant species such as *Arabidopsis* (75 bZIPs), rice (89 bZIPs), and sesame (62 bZIPs), perilla contains more bZIP genes (101), indicating a significant expansion of the *PfbZIP* gene family. In order to explain the potential mechanism responsible for this expansion, we employed TBtools 2.0 software to investigate the duplication pattern of *PfbZIP* genes, which contain both tandem and segmental duplication. As shown in Figure 3A and Table S3, a high degree of homology was detected among *PfbZIP* genes across different chromosomes despite no tandem duplication. Totally, 83 pairs of segmental duplication genes were detected in the *PfbZIP* gene family, and the number of replicates of *PfbZIP* genes with segmental duplication varied from one to four. No duplication was detected for 14 *PfbZIP* genes, including *PfbZIP7*, *PfbZIP11*, *PfbZIP13*, *PfbZIP14*, *PfbZIP27*, *PfbZIP29*, *PfbZIP38*, *PfbZIP41*, *PfbZIP49*, *PfbZIP55*, *PfbZIP72*, *PfbZIP76*, *PfbZIP81*, and *PfbZIP89*. This result indicates that segmental duplication may serve as the core driving force in the evolution and amplification of the *PfbZIP* gene family.

To deeply investigate the origin and phylogenetic evolution of *PfbZIP* genes, systematic comparative analysis was conducted between *P. frutescens*, the model plant *Arabidopsis*, and its closely related plant species, *S. indicum*. As illustrated in the comparative synteny maps (Figure 3B and Tables S4 and S5) between *P. frutescens* and the other two species (*Arabidopsis* and *S. indicum*), the corresponding relationships were identified between 52 *PfbZIP* genes and 38 *AtbZIP* genes, and the corresponding 78 pairs of orthologous *bZIP* genes were built between perilla and *Arabidopsis*. Remarkably, some *PfbZIP* genes were recognized as potential orthologs of a specific *AtbZIP* gene. For instance, *PfbZIP8*, *PfbZIP15*, *PfbZIP65*, and *PfbZIP91* were found to be orthologs of *AtbZIP48*. Furthermore, some *PfbZIP* genes exhibited collinearity relationships with multiple *AtbZIP* genes. For example, *PfbZIP65* was identified as the ortholog of *AtbZIP39*, *AtbZIP41*, and *AtbZIP48* (Figure 3B and Table S4). For comparison between perilla and sesame, the corresponding relationships were also identified between 92 *PfbZIP* genes and 63 *SibZIP* genes, and then 148 pairs of orthologous *bZIP* genes were established between these two plant species (Figure 3B and Table S5).

### 2.4. Functional Classification of PfbZIP Genes by GO and KEGG

**Figure 4:**
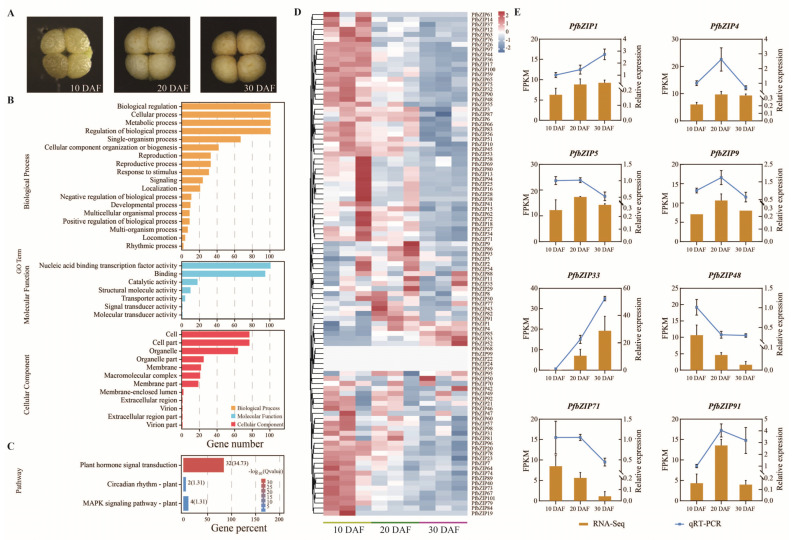
GO and KEGG enrichment analysis and expression profiles of *PfbZIP* genes during perilla seed development. (**A**) The distinct seed developmental stages of the perilla variety ‘Jinzisu 1’. (**B**) GO enrichment analysis of *PfbZIP* gene family members. (**C**) KEGG enrichment analysis of *PfbZIP* gene family members. (**D**) Heat map of the *PfbZIP* gene expression patterns in developing seeds of *P. frutescens*. (**E**) The relative expression levels of the selected *PfbZIP* genes were detected by RT-qPCR in seed development. Values are presented as mean ± SD (*n* = 3).

The gene ontology (GO) annotation examination indicated that 101 PfbZIP proteins were grouped into three GO terms: biological process (BP), molecular function (MF), and cellular component (CC) (Figure 4B). In biological process, PfbZIP proteins were mainly involved in 18 categories, with predominant function in response to biological regulation (GO: 0065007), followed by cellular process (GO: 0009987), metabolic process (GO: 0008152), as well as regulation of biological process (GO: 0050789) and single-organism process (GO: 0044699). In molecular function, PfbZIPs mainly acted as nucleic acid-binding transcription factor activity (GO: 0001071) and binding activity (GO: 0005488), followed by catalytic activity (GO: 0003824), structural molecule activity (GO: 0005198), and transporter activity (GO: 0005215). With regard to the cellular component category, PfbZIPs were largely annotated into cell (GO: 0005623), cell part (GO: 0044464), and organelle (GO: 0043226), followed by organelle part (GO: 0044422), membrane (GO: 0016020), macromolecular complex (GO: 0032991), and membrane part (GO: 0044425).

Pathway-based analysis was performed to explore the potential roles of the *PfbZIP* genes. A total of 38 *PfbZIP* genes were distributed across three pathways in the KEGG database, including circadian rhythm-plant (ko04712), plant signal transduction (ko04075), and MAPK signaling pathway-plant (ko04016) (Figure 4C). The results illustrate that PfbZIPs may mediate the regulation of diverse life processes in perillas.

### 2.5. Analysis of the Expression Patterns of the PfbZIP Genes during Perilla Seed Development

To explore the transcriptional patterns and potential roles of *PfbZIP* genes in perilla seed development, the expression levels (FPKM values) of *PfbZIP* genes were analyzed in three distinct developmental stages (10, 20, and 30 days after flowering, DAF, Figure 4A) of seeds using transcriptome data obtained in our lab. As shown in the heatmap of *PfbZIP* gene expressions (Figure 4D), different *PfbZIP* genes exhibited diverse expression patterns during perilla seed development. Interestingly, a greater number of *PfbZIP* genes exhibited downregulation compared to those showing upregulation. Specifically, a progressively decreased pattern of expression was detected for 59 *PfbZIP* genes, including *PfbZIP7*, *PfbZIP10*, *PfbZIP12*, *PfbZIP13*, *PfbZIP16*, and so on. On the other hand, *PfbZIP1*, *PfbZIP33*, *PfbZIP52*, and *PfbZIP95* showed an increasing trend in expression. Notably, *PfbZIP3*, *PfbZIP18*, *PfbZIP29*, *PfbZIP30*, *PfbZIP54*, and *PfbZIP85* exhibited relatively high expression levels at 10 and 20 DAF, then significantly reduced at 30 DAF. Whereas, *PfbZIP22*, *PfbZIP24*, *PfbZIP39*, *PfbZIP68*, and *PfbZIP99* were barely expressed during the development of perilla seeds.

To further investigate the function of *PfbZIP* genes, RT-qPCR analysis was employed to assess the expression patterns of *PfbZIP* genes in developing perilla seeds. The expression abundances of the eight selected *PfbZIP* genes were analyzed in the developmental seeds of ‘Jinzisu 1’ (Figure 4E). These eight *PfbZIP* genes exhibited differential expression patterns across various developmental stages of seeds. In accordance with the transcriptome data, the relative expression levels of the *PfbZIP4*, *PfbZIP5*, *PfbZIP9*, and *PfbZIP91* genes exhibited an initial fast increase and then a subsequent decrease during seed development. The levels of *PfbZIP48* and *PfbZIP71* gene expressions exhibited a gradual reduction during seed development. In contrast, the expression levels of the *PfbZIP1* and *PfbZIP33* genes gradually increased. Notably, the expression abundance of the *PfbZIP33* gene was found to be much higher than that of other *PfbZIP* members. These findings suggest that most *PfbZIP* genes possess constitutive functionality in perilla seed development, with a number of them acting differentially across distinct seed developmental stages.

### 2.6. Identification of Candidate PfbZIP TFs Mediating Transcription and Regulation of Enzyme Genes Involved in Lipid Metabolism

The bZIP TF can selectively bind to the A/C/T/CACGTG/C/T/A or GCCACGTGGA motif sequences, thereby activating or inhibiting expression of the target genes [22,25]. To ascertain whether PfbZIP TFs regulate transcription of lipid-associated genes, *cis*-elements in the promoter sequences of lipid synthesis-related genes in perilla were examined through the Plantcare website to detect the bZIP-binding *cis*-elements in these selected genes. As shown in Table 1, the bZIP-binding motif sequences (ACGT) were identified in the promoter regions of eight genes associated with lipid metabolism. These eight lipid-related genes contained three de novo FA synthesis enzymes (*PfKAS2*, *PfSAD2*, and *PfFAD6*) three FA desaturases, and five acyltransferases (*PfGPAT1*, *PfLPAT1B*, *PfDGAT2A*, *PfDGAT3A*, and *PfPDAT2B*). This *cis*-element analysis indicates that these eight lipid-related genes may be regulated by PfbZIP TFs.

**Figure 5:**
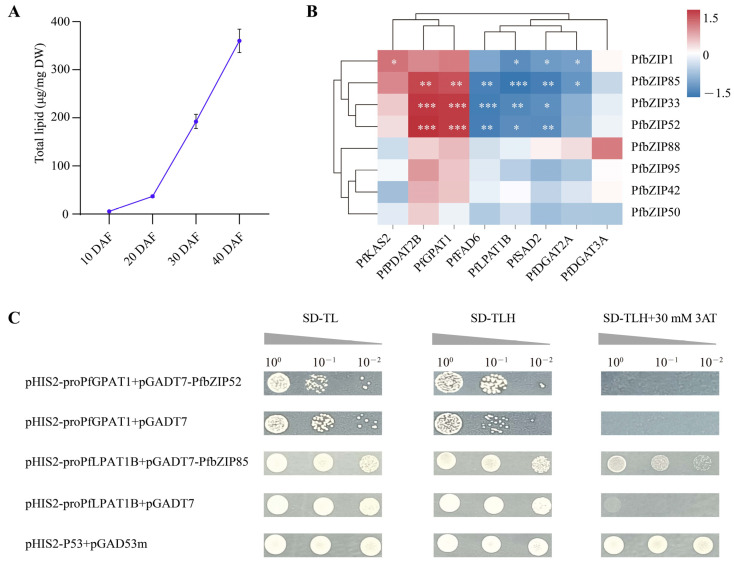
Prediction and interaction analysis of candidate PfbZIP TF-regulated target genes involved in lipid biosynthesis. (**A**) Total lipid dynamic accumulation in the developing seed of perilla. The total lipid contents are expressed by the mass of esterified fatty acids. (**B**) Heat map of expression correlation between eight PfbZIP TFs and key lipid-related enzyme genes. Red indicates a positive correlation, while blue indicates a negative correlation. * represents a significant difference at *p* < 0.05. ** represents a significant difference at *p* < 0.01. *** represents a significant difference at *p* < 0.001. (**C**) Yeast one-hybrid assay of PfbZIP52 and PfbZIP85 with promoters of the *PfGPAT1* and *PfLPAT1B* genes, respectively.

Dynamic analysis was conducted for total lipid accumulation during perilla seed development. As shown in Figure 5A, total lipid content was just 5.79 μg/mg at 10 DAF and then started a slow increase to 36.88 μg/mg at 20 DAF and sharply enriched to 192.45 μg/mg at 30 DAF, followed by the peak level of 360.15 μg/mg at 40 DAF (the seed almost matured). In accordance with this dynamic of total lipid accumulation (Figure 5A), the expression levels of *PfbZIP1*/*33*/*42*/*50*/*52*/*85*/*88*/*95* (Figure 4D) were significantly upregulated throughout the fast accumulation stage of total lipid in perilla seeds, indicating that these eight PfbZIPs may function importantly for the lipid/FA biosynthesis pathway in perilla seeds.

Therefore, we further investigated the expression correlation between these eight PfbZIP TFs and the aforementioned eight key lipid-related enzyme genes. As shown in Figure 5B, the expressions of both *PfbZIP52* and *PfbZIP33* exhibited a significantly positive correlation (*p* < 0.001) with the expressions of the *PfPDAT2B* and *PfGPAT1* genes. However, the expression of *PfbZIP33* displayed a strong negative correlation (*p* < 0.001) with the expression of the *PfFAD6* gene. Remarkably, *PfbZIP85* displayed an extremely negative association (*p* < 0.001) with *PfLPAT1B*. Such expression correlation analysis demonstrates that *PfPDAT2B* and *PfGPAT1* genes may be the downstream targets of PfbZIP52 and PfbZIP33 TFs, while the *PfLPAT1B* gene may be the target of PfbZIP85. Moreover, this suggests that these three PfbZIP TFs might be the candidate bZIP TFs directly regulating the transcription levels of the lipid genes in perilla, thereby mediating FA/oil biosynthesis and accumulation.

### 2.7. PfbZIP85 Rather than PfbZIP52 Physically Interacts with Promoters of the Downstream Target Genes Revealed by the Yeast One-Hybrid Assay

To verify whether the candidate PfbZIPs directly regulate the downstream target genes, a yeast one-hybrid (Y1H) assay was employed to investigate the physical interaction between PfbZIP52/85 and the promoters of the *PfGPAT1*/*PfLPAT1B* genes, respectively. The CDS of *PfbZIP52* and *PfbZIP85* genes were isolated from the perilla variety ‘Jinzisu 1’ (Figure S2) and then separately inserted into the prey vector pGADT7 to form pGADT7-PfbZIP52 and pGADT7-PfbZIP85, respectively (Figure S3). At the same time, the ACGT element-containing promoter sequences of *PfGPAT1* and *PfLPAT1B* genes were isolated and separately inserted into the bait vector pHIS2 to generate pHIS2-proPfGPAT1 and pHIS2-proPfLPAT1B, respectively (Figure S3).

For this assay, the bait vectors pHIS2-proPfGPAT1 and pHIS2-proPfLPAT1B were co-transformed into the Y187 yeast strain with the effector plasmid pGADT7, respectively. Meanwhile, pHIS2-P53 and pGAD53m plasmids were co-transformed into Y187 as the positive controls. The results showed that the positive control yeast strains grew normally on SD/-Trp/-Leu/-His (SD-TLH) medium added with different concentrations of 3-amino-1,2,4-triazole (0, 10, 20, 30, 40, 50, 75, and 100 mmol/L 3AT). However, the co-transformed strains (pHIS2-proPfGPAT1 + pGADT7 and pHIS2-proPfLPAT1B + pGADT7) failed to grow on SD-TLH medium supplemented with 30 mmol/L 3AT (Figure S4). This indicates that neither *PfGPAT1* nor *PfLPAT1B* promoters are self-activated.

Therefore, SD-TLH medium supplemented with 30 mmol/L 3AT was used in Y1H to further investigate the interaction between PfbZIP52 and the *PfGPAT1* promoter, as well as PfbZIP85 and the *PfLPAT1B* promoter, respectively. As shown in Figure 5C, the positive control pHIS2-P53 + pGAD53m exhibited normal growth on all the tested media, including SD-TL, SD-TLH, and SD-TLH + 30 mmol/L 3AT. However, the growth of the control groups, pHIS2-proPfGPAT1 + pGADT7 and pHIS2-proPfLPAT1B + pGADT7, was inhibited on SD-TLH + 30 mmol/L 3AT medium. Remarkably, the yeast strains co-expressed with pHIS2-proPfLPAT1B + pGADT7-PfbZIP85 grew normally on the medium of SD-TLH + 30 mmol/L 3AT, while the yeast co-expressed with pHIS2-proPfGPAT1 + pGADT7-PfbZIP52 failed to grow. Taken together, no interaction was observed between the *PfGPAT1* promoter and PfbZIP52 TF. Notably, PfbZIP85 TF can physically interact with the promoter of the *PfLPAT1B* gene, suggesting that PfbZIP85 TF may directly modulate the expression of the downstream target gene *PfLPAT1B*, one of the key genes for TAG assembly.

### 2.8. PfbZIP85 Is Localized in the Nucleus and Negatively Regulates the Expression of the PfLPAT1B Gene

**Figure 6:**
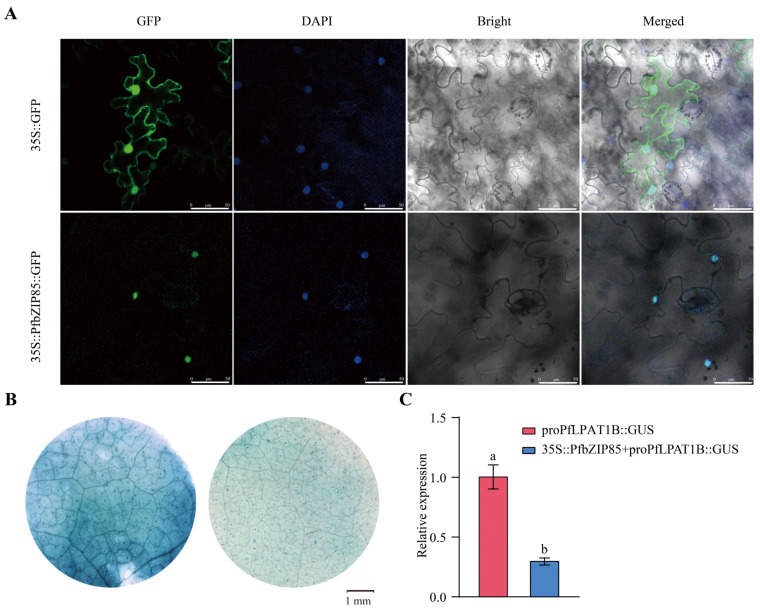
(**A**) Subcellular localization of PfbZIP85 in tobacco leaf cells. The fluorescence signals were observed using a laser-scanning confocal microscope (Leica TCS SP8). Scale bars = 50 μm. (**B**) GUS staining in the infiltrated tobacco leaves. Left, tobacco leaves infiltrated with the proPfLPAT1B::GUS plasmid alone. Right, tobacco leaves co-infiltrated with 35S::PfbZIP85 and proPfLPAT1B::GUS plasmids. (**C**) Expression analysis of the *GUS* gene in the transiently infected tobacco leaves. Values are presented as mean ± SD (*n* = 3). ‘a’ and ‘b’ indicate significant differences at *p* < 0.05.

To further characterize the PfbZIP85 TF, we constructed a recombinant fusion plasmid (35S::PfbZIP85::GFP) (Figure S3) and analyzed the subcellular localization of the PfbZIP85 protein. This vector containing PfbZIP85 fused with GFP was transiently expressed in *N. benthamiana* leaves, while the empty vector pCAMBIA1300/GFP was used as the negative control. As observed in Figure 6A, the green fluorescence signal from the 35S::GFP fusion vector was exhibited in both the nucleus and cytoplasm membrane, while the 35S::PfbZIP85::GFP fusion vector generated a green fluorescence signal only in the nucleus of the cell, confirming that PfbZIP85 TF is a nuclear-localized protein.

Subsequently, a GUS assay was used to identify the regulatory mechanism of PfbZIP85 TF on the *PfLPAT1B* gene. The promoter (2000 bp in length) of the *PfLPAT1B* gene (Figure S5) was fused with the GUS report gene of the pCAMBIA1301 vector to form a fusion expression vector of pro*PfLPAT1B* (pro*PfLPAT1B*::GUS) (Figure S3). The pro*PfLPAT1B*::GUS plasmid alone or with 35S::PfbZIP85 was transiently expressed in tobacco leaves. The tobacco plants cultured for 36 h to 48 h were taken for GUS staining detection. As observed in Figure 6B, the leaves co-infiltrated with 35S::PfbZIP85 and pro*PfLPAT1B*::GUS displayed a lighter blue color (GUS activity) than those infiltrated with pro*PfLPAT1B*::GUS alone. Further RT-qPCR examination of the relative expression level of the GUS gene in tobacco leaf samples revealed that the GUS expression in the leaves co-infiltrated with 35S::PfbZIP85 and pro*PfLPAT1B*::GUS was significantly reduced by 70.12% (*p* < 0.05) compared to the leaves infiltrated with pro*PfLPAT1B*::GUS alone (Figure 6C). Collectively, these results demonstrate that PfbZIP85 exerts a negative regulatory role on the expression of the *PfLPAT1B* gene.

### 2.9. Heterogeneous Expression of PfbZIP85 Resulted in a Reduction of TAG Content and an Alternation of TAG-Associated FAs in Transgenic Tobacco Lines

To further evaluate the functions of PfbZIP85 and its potential as a target in biological engineering to enhance the production and quality of vegetable oils/TAG, plant constitutive expression vectors and seed-specific expression vectors of the *PfbZIP85* gene were constructed (Figure S3) and subsequently transformed into common tobacco, respectively. The wild-type (WT) tobacco plants were employed as the negative controls. The *PfbZIP85*-expressed plants were identified (Figure S6), and then three independent transgenic lines exhibiting higher expression of *PfbZIP85* were used as the samples for phenotypic assessment, including TAG content, FA profiles, as well as other agronomic characteristics.

**Figure 7:**
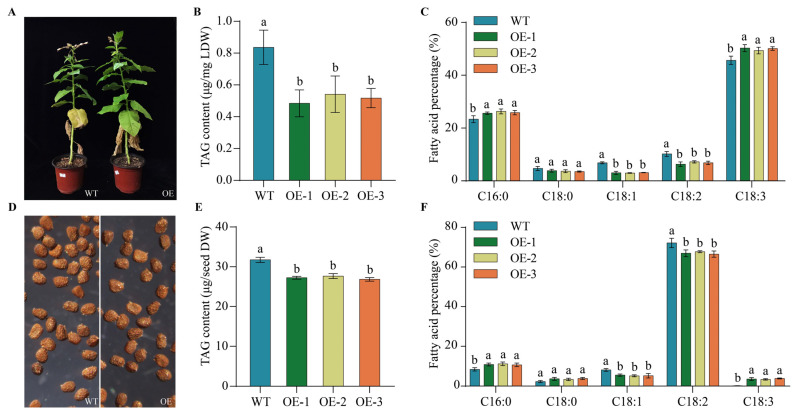
Analysis of TAG content and major FAs in TAG in the transgenic tobacco lines. (**A**) Transgenic tobacco plants. (**B**,**C**) Contents of total TAG and key TAG-associated FAs in *PfbZIP85*-expressing tobacco leaves. (**D**) Transgenic tobacco seeds. (**E**,**F**) Contents of total TAG and key TAG-associated FAs in *PfbZIP85*-expressing tobacco seeds. LDW, the dry weight of tobacco leaves. WT, untransformed tobacco plant. OE-1/-2/-3, the transgenic tobacco lines overexpressing the *PfbZIP85* gene. The TAG contents are expressed by the mass of esterified fatty acids. Values are presented as mean ± SD (*n* = 3). Different lowercase letters indicate significant differences at *p* < 0.05.

Total lipid was extracted from leaves (Figure 7A) or seeds (Figure 7D) from tobacco plants overexpressing *PfbZIP85*, followed by the preparation of FAMEs for measuring the contents of total oil and TAG-associated FAs through GC (see the Section 4 for details). As displayed in Figure 7B, the TAG content in the leaves was significantly decreased from 0.84 μg/mg in the WT lines to 0.51 μg/mg in PfbZIP85-expressing lines (*p* < 0.05). Additionally, the TAG-associated FA profiles were identical between the WT and *PfbZIP85*-expressing tobacco leaves (Figure 7C). *PfbZIP85*-expressing lines indicated a significant increase in the levels of C18:3 and C16:0 by 2.54% and 4.25% (*p* < 0.05), respectively, compared to the WT lines in leaf tissues. Correspondingly, the levels of C18:1 and C18:2 were both notably decreased by 3.78% and 3.42% (*p* < 0.05) in the *PfbZIP85*-expressing tobacco leaves, and the C20:0 exhibited a small reduction compared to the WT lines (Figure 7C). Similarly, the TAG content in the seeds was significantly decreased from 31.74 μg/seed in the WT lines to 27.25 μg/seed in PfbZIP85-expressing lines (*p* < 0.05) (Figure 7E). FA profiles in TAGs from tobacco seeds (Figure 7F) also indicated the same alteration trend as the case in leaves, with contents of C18:3 and C16:0 notably enhanced by 3.42% and 2.50% (*p* < 0.05), respectively, followed by a remarkable reduction of C18:1 and C18:2 contents (*p* < 0.05) in transgenic tobacco seeds compared to the WT seeds (Figure 7F). These results indicate that PfbZIP85 can function efficiently in the ectopic host, affecting the TAG level and ratio of major TAG-associated FAs in plant seeds or non-seed tissues.

### 2.10. Overexpression of PfbZIP85 Results in No Significantly Negative Impacts on Other Agronomic Traits of Tobacco Plants

**Figure 8:**
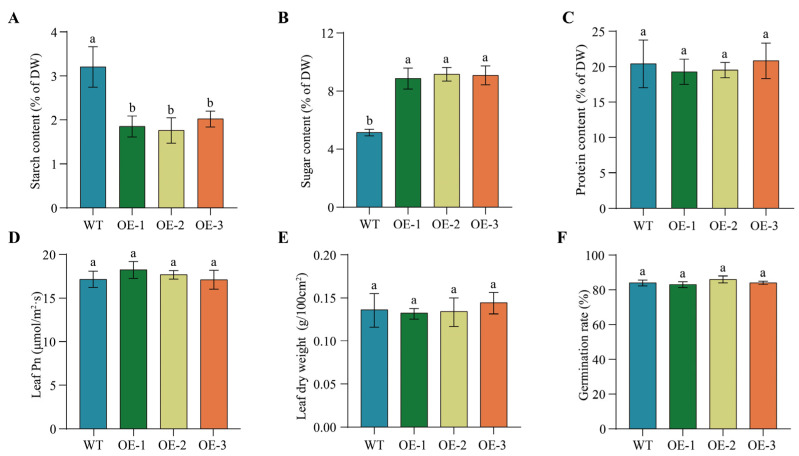
Other agronomic traits in tobacco plants overexpressing the *PfbZIP85* gene. (**A**) Starch content. (**B**) Soluble sugar content. (**C**) Protein content. (**D**) Leaf photosynthesis (Pn). (**E**) Leaf dry weight. (**F**) Seed germination rate. WT, untransformed tobacco plant. OE-1/-2/-3, the transgenic tobacco lines overexpressing the PfbZIP85 gene. Values are presented as mean ± SD (*n* = 3). Different lowercase letters indicate significant differences between different samples at *p* < 0.05.

To examine whether the overexpression of *PfbZIP85* affects other physiological characteristics in the heterogeneous host, we measured the contents of starch, soluble sugar, and protein, leaf photosynthesis (Pn), leaf dry weight (LDW), thousand seed weight (TSW), and seed germination rate. As illustrated in Figure 8A, the starch content in seeds was significantly decreased from 3.20% in the WT lines to 1.88% in *PfbZIP85*-expressing lines (*p* < 0.05). Whereas, soluble sugar content exhibited a significant enhancement of 3.89% (*p* < 0.05) in the *PfbZIP85*-expressing lines compared to WT lines (Figure 8B). Protein content in tobacco leaves displayed no obvious difference between the WT lines and the transgenic plants (Figure 8C). In addition, no apparent distinctions were examined for leaf Pn (Figure 8D), LDW (Figure 8E), or germination rate (Figure 8F) among all the tested samples. In short, these findings illustrate that the expression of *PfbZIP85* in tobacco can notably reduce TAG, C18:1, and C18:2 levels but increase C18:3 content without a significant penalty on other agronomy traits despite the increased starch level. Speculatively, down-regulation of *PfbZIP85* might increase TAG accumulation and C18:1 and C18:2 contents in perilla seeds.

### 2.11. Overexpression of PfbZIP85 Affects the Expression of Genes Associated with Lipid Metabolism in Tobacco Plants

**Figure 9:**
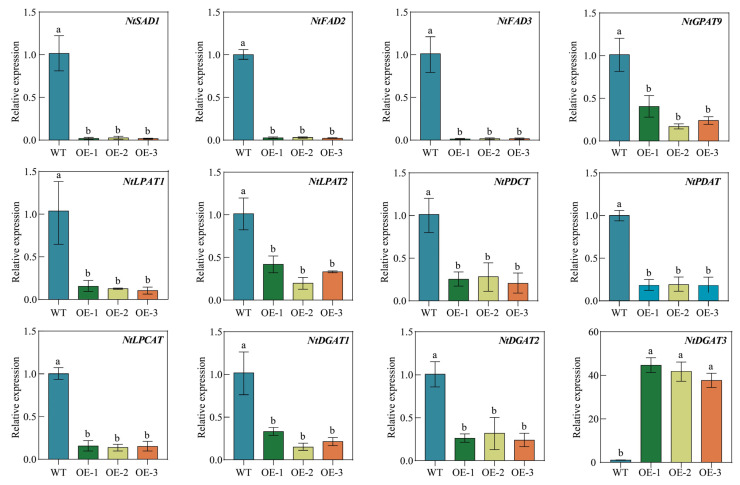
Expression analysis of genes associated with FA/oil biosynthesis in the transgenic tobacco plant overexpressing *PfbZIP85*. The *N. tabacum actin* gene was used as the internal control gene. WT, untransformed tobacco plant. OE-1/-2/-3, the *PfbZIP85*-expressing tobacco lines. Values are presented as mean ± SD (*n* = 3). Different lowercase letters indicate significant differences between different samples at *p* < 0.05.

To further investigate the potential molecular mechanism of PfbZIP85 in heterogeneous host tobacco plants, we examined the expression patterns of the twelve lipid genes in the transgenic tobacco plants overexpressing *PfbZIP85*. The tested twelve genes included the genes encoding Δ^9^ stearoyl-ACP desaturase (SAD1), fatty acid desaturases (FAD2 and FAD3), glycerol-3-phosphate acyltransferase (GPAT9), lysophosphatidic acid acyltransferases (LPAT1 and LPAT2), phosphatidylcholine diacylglycerol choline phosphotransferase (PDCT), phospholipid diacylglycerol acyltransferase (PDAT), lysophosphatidylcholine acyltransferase (LPCAT), and diacylglycerol acyltransferases (DGAT1, DGAT2, and DGAT3), respectively. As shown in Figure 9, the expression levels of *NtSAD1*, *NtFAD2*, *NtFAD3*, *NtGPAT9*, *NtLPAT1*, *NtLPAT2*, *NtPDCT*, *NtPDAT*, *NtLPCAT*, *NtDGAT1*, and *NtDGAT2* genes were significantly decreased in *PfbZIP85*-expressing tobacco plants compared with WT plants. Remarkably, the expression level of *NtDGAT3* was significantly increased, exhibiting a 40.32-fold enhancement compared to the WT plants (Figure 9). The down-regulation of these endogenous genes responsible for FA/oil biosynthesis might contribute to the decrease in TAG accumulation in the transgenic tobacco tissues. Moreover, PfbZIP85 can negatively regulate *NtLPAT1*/*2* expressions and possibly down-regulate the expressions of other lipid-related genes (*NtSAD1*, *NtFAD2*, *NtFAD3*, and so on), except for increasing *NtDGAT3* expression. Collectively, these findings demonstrate that the PfbZIP85 protein has the potential to modulate FA/oil biosynthesis and accumulation by regulating the expressions of lipid biosynthesis-related genes.

## 3. Discussion

*Perilla frutescens* is a specific oilseed crop because of its nutritional and medicinal properties, with its seed containing 46–58% oil [56]. Perilla seed oil is rich in UFAs (more than 90%), with a high level of α-linolenic acid (>65%) [2]. Therefore, an in-depth analysis of the biosynthesis and regulatory mechanisms of ALA-enriched oil in perilla seed would provide valuable insights for improving the oil yield and quality of perilla seed and other oilseeds, thereby meeting the growing market demand for nutrient-healthy vegetable oils. A number of TFs were identified to mediate regulation of multiple life processes, including FA/oil biosynthesis in plants [49,50,51,52,53]. Of them, bZIP TFs were found to play a key regulatory role in various plants [30,57,58,59]. However, perilla PfbZIP members and their functions remain unknown yet. In the present study, a genome-wide identification of perilla PfbZIP was performed, followed by functional characterization of PfbZIP85 through yeast one-hybrid (Y1H) assays and tobacco genetic transformations, aiming to enhance our comprehension of PfbZIP roles in FA/oil metabolism and also providing valuable bZIP TFs as targets for plant lipid metabolic engineering.

### 3.1. Characteristics and Evolution of PfbZIP Family Members

In this study, 101 PfbZIP members (PfbZIP1-PfbZIP101) were obtained in the *P. frutescens* genome, showing an uneven distribution across chromosomes (Figure S1). Sequence annotation revealed that the average length of PfbZIP proteins is 342 aa, which is larger than that in other reported plants such as *Arabidopsis* (321 aa) [26], castor bean (326 aa) [25], rapeseed (292 aa) [31], soybean (343 aa) [32], and sesame (339 aa) [33].

All these 101 PfbZIP proteins were divided into 11 groups (groups A, B, C, D, E, F, G, H, I, S, and UC) (Figure 1). Especially, nine PfbZIP proteins (PfbZIP14, 19, 22, 24, 38, 41, 49, 68, and 76) were classified into the UC group, suggesting they might be new members generated during the evolution of the PfbZIP family. Multiple protein sequence alignment analyses disclosed that except for the PfbZIP members within the UC group, all other PfbZIP proteins contained a highly conserved basic region (N-x7-R/K) at the N-terminus, which is essential for DNA binding and nuclear localization (Figure 2) [33]. Moreover, the C-terminus of these PfbZIP proteins had a highly conserved leucine zipper region (L-x6-L-x6-L), which is related to specific recognition and dimerization of PfbZIP proteins (Figure 2) [26,27]. Additionally, the same conserved motifs and a high degree of sequence similarity among PfbZIP members in the same group suggest potential functional redundancy.

Gene duplication played a crucial role in gene family expansion and genome evolution [49,60]. In our study, 83 pairs of segmental duplicated *PfbZIP* genes were observed, accounting for approximately 82.2% of the *PfbZIPs*. Meanwhile, no tandem duplications were discovered among the *PfbZIP* genes (Figure 3A). Therefore, segmental duplication plays a predominant role in the expansion and evolution of the *PfbZIP* gene members in perilla, which is consistent with previous findings in rice [27], sesame [33], and grapevine [61]. Previous studies have demonstrated that gene duplication leads to the divergence of spatiotemporal expression patterns of genes [62,63,64]. Thus, we further analyzed the expression profiles of all *PfbZIP* members in the developing seeds of perilla. The results showed that these 83 pairs of *PfbZIP* genes exhibited various expression patterns (Figure 4D) across different developmental stages of perilla seeds, indicating that functional divergence occurred in these gene pairs during evolution. The difference in expression levels among PfbZIP members might be the fundamental cause of their functional differences. In addition, 101 *PfbZIP* genes were enriched in three GO functional categories: BP, MF, and CC (Figure 4B) by GO analysis, and 38 of the 101 *PfbZIP* genes were enriched in three pathways (e.g., plant signal transduction, circadian rhythm-plant, and MAPK signaling pathway-plant) (Figure 4C) by KEGG analysis. Both GO and KEGG analyses indicate that PfbZIPs may mediate signal transduction processes in perilla plants (e.g., abscisic acid (ABA) and jasmonic acid (JA) signal transduction pathways).

### 3.2. PfbZIP Members May Play Significant Roles in Lipid Biosynthesis by Regulating the Expression of Lipid-Associated Genes

Transcription factors regulate the transcriptional level of other transcription factors or their downstream target genes via binding with DNA *cis*-acting elements [22,25]. Despite a number of bZIP TFs being identified in various plants and their diverse functions being reported [36,48,65], only a few bZIP members were detected to mediate lipid metabolism. To identify whether PfbZIP members regulate FA/oil metabolism in perilla seeds, expression profiles of *PfbZIP* genes were examined during perilla seed development by RNA-Seq and RT-qPCR. As shown in Figure 4D,E, different *PfbZIP* members exhibited distinct expression patterns at different developmental stages of perilla seeds, suggesting that different PfbZIP members may function diversely during seed development. Furthermore, the expression abundances of the eight *PfbZIP* members (*PfbZIP1*/*33*/*42*/*50*/*52*/*85*/*88*/*95*) were obviously upregulated in the rapid oil-enrichment period in perilla seeds (Figure 4D and Figure 5A), indicating that these PfbZIP members may function crucially in the regulation of FA/oil accumulation in perilla seeds. 

Interestingly, one or more bZIP-binding *cis*-elements (ACGT) were present in the promoters of the eight key FA/oil-related enzyme genes (*PfKAS2*, *PfSAD2*, *PfFAD6*, *PfGPAT1*, *PfLPAT1B*, *PfDGAT2A*, *PfDGAT3A*, and *PfPDAT2B*) (Table 1), demonstrating that these eight enzyme genes may be regulated by PfbZIP TFs. Subsequently, the expression correlation analysis indicated that the expression of some *PfbZIP* genes was significantly correlated with the oil-related enzyme genes (Figure 5B). For instance, a highly significant positive correlation was observed between the expression of *PfbZIP52* and *PfGPAT1*, whereas a highly remarkable negative correlation was detected between the expression of *PfbZIP85* and *PfLPAT1B* (Figure 5B). These data further indicate that PfbZIP might mediate FA/oil biosynthesis by regulating the expressions of the lipid genes in perilla developmental seeds. Finally, the Y1H test and GUS activity assay evidenced that PfbZIP85 TF negatively regulated *PfLPAT1B*’s expression by physical interaction with the promoter of the *PfLPAT1B* gene (Figure 5C and Figure 6B,C). In accordance with the regulatory function of PfbZIP85 identified here, AtbZIP67 in *Arabidopsis* [50] and RcbZIP in castor bean [25] were reported to bind to the ACGT elements and then modulate the transcriptional activity of their respective downstream target genes. 

### 3.3. PfbZIP85 Can Be Used as a Genetic Modification Target to Enhance Oil Production and Quality in Plant Seed or Non-Seed Tissues

The bZIP TF family is one of the important families in the plant kingdom [66,67], mainly participating in the modulation of plant growth and development, organ differentiation [36,37,38], energy metabolism homeostasis [39,40,41], transcriptional control of genes associated with seed storage substances [42], abiotic stress response [43,44,45,46,47,48], as well as FA/oil biosynthesis and accumulation [49,50,51,52,53]. For example, AtbZIP67 specifically binds to the G-box element within the *AtFAD3* promoter region and improves the biosynthesis of C18:3 in transgenic *Arabidopsis* seeds [50]. Overexpression of the *GmbZIP123* gene in *Arabidopsis* improved oil content in transgenic plant seeds [51]. Furthermore, overexpression of *NsbZIP1* from *N. salina* algae CCMP1776 leads to an upregulation of the transcription of lipid genes including *ACBP*, *KAS*, *LC-FACS*, and *LPAAT* in algal cells under nitrogen stress, along with a significant improvement of neutral lipid contents [53]. However, bZIP TFs can also inhibit plant lipid synthesis and accumulation. For instance, overexpression of AtbZIP52 resulted in a decrease in seed oil level in the transgenic plant lines, whereas an enhancement of seed oil accumulation was observed in the *Atbzip52* loss-of-function mutant lines compared with WT *Arabidopsis* [67]. 

In this study, common tobacco was used as the heterogeneous host to overexpress the *PfbZIP85* gene to assess the potential of PfbZIP85 in genetic engineering for regulating TAG accumulation and FA profiles in both leaves and seeds. Our findings exhibited a significant reduction of TAG content in the transgenic tobacco leaves (Figure 7B). Similarly, a reduction was observed for TAG content in the transgenic tobacco seeds (Figure 7E). Moreover, the ratio of FA compositions in TAGs was also changed within *PfbZIP85*-transgenic tobacco leaves and seeds, showing significant increases in C18:3 and C16:0 contents, accompanied by remarkable decreases in the levels of C18:1 and C18:2 (Figure 7C,F). For the analysis of TAG-associated FAs, the accuracy may be improved by using other non-destructive reagents (2,7-dichlorofluorescein, rhodamine, or primulin) for displaying silica gel zones on TLC containing lipids, instead of iodine vapor (an oxidizing agent), which may damage lipids, particularly polyunsaturated fatty acids. 

**Figure 10:**
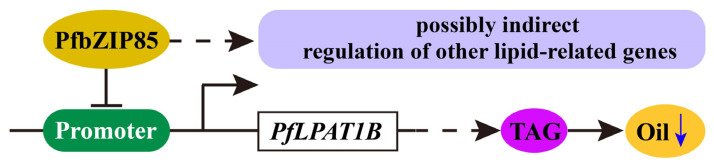
Mechanistic model of FA/TAG biosynthesis mediated by the PfbZIP85 TF. For simplicity, only one TAG molecule is displayed, and certain intermediates or reactions are omitted. The blue downward arrow indicates the reduction in oil content.

In contrast to the reduction of both total oil and starch contents (Figure 7B and Figure 8A), overexpression of *PfbZIP85* led to an increase in soluble sugar content (Figure 8B), while the protein content (Figure 8C) remained unchanged. These findings suggest that PfbZIP85 inhibits carbon flux into the FA/oil pathway but increases carbon flux to store soluble sugar in the tissue. Interestingly, no negative effect was observed on other agronomic traits, including leaf Pn, leaf dry weight, and germination rate (Figure 8D–F), in the transgenic tobacco lines. Further gene expression analysis uncovered that overexpression of *PfbZIP85* resulted in an expression change of the 12 lipid biosynthesis enzyme genes in the transgenic tobacco lines (Figure 9), showing a reduced expression of the 11 endogenous enzyme genes but an increased expression of the *NtDGAT3* gene compared to the WT controls. Taken together, PfbZIP85 from perilla can be recognized as a target in lipid metabolic engineering to regulate oil yield and target FA enrichment in the heterogeneous plant host, despite the detailed regulation mechanism of FA/oil biosynthesis and accumulation mediated by PfbZIP85 or its homolog in different plants needing further investigation. Based on these findings, therefore, we proposed a possible regulatory mechanism for FA/oil metabolism mediated by PfbZIP85 TF in plant tissues (Figure 10).

Recently, AtbZIP52 was found to interact with AtWRI1, a core transcriptional regulator in the lipid biosynthesis pathway, leading to the down-regulation of several fatty acid biosynthesis genes and thus reduced oil production in Arabidopsis seeds [67]. MicroRNA167 was identified to target the CsARF8 transcription factor in *Camelina sativa*, and CsARF8 upregulated the expression of the *CsFAD3* gene by cooperating with bZIP67 and ABI3, thereby affecting ALA biosynthesis in *C. sativa* seeds [68]. Increasing data indicated that plant seed oil biosynthesis and accumulation were controlled by a complex and multi-level regulatory mechanism, particularly a network of interacting transcriptional activators and repressors [69]. Our study identified PfbZIP85 as a negative regulator in perilla seed oil biosynthesis, showing that PfbZIP85 can be a target in genetic engineering for the improvement of oil yield and quality in heterogenous hosts. Further research needs to address whether other TFs or miRNAs and even epigenetic regulators interact with PfbZIP85 and to identify more target genes of PfbZIP85, which will deepen our understanding of the PfbZIP85-mediated internet responsible for FA/oil biosynthesis during seed development and other biological processes.

## 4. Materials and Methods

### 4.1. Biological Materials

The high-oil-content perilla (*Perilla frutescens*) variety ‘Jinzisu 1’ was selected for this study and grown in Taigu District, Shanxi Province, China (112°58′ E, 37°42′ N). Seeds of ‘Jinzisu 1’ were harvested at 10, 20, and 30 days after flowering (DAF). All samples were promptly flash-frozen using liquid nitrogen and subsequently stored at −80 °C for further experiments. The tobacco species *Nicotiana benthamiana* was employed for protein subcellular localization in this study, while the common tobacco species *Nicotiana tabacum* was utilized for gene transformation. The growth conditions of all the tobacco plants included a photoperiod of 16 h of light and 8 h of darkness at 25 °C [6].

The bacterial and yeast strains, as well as the plasmids used in this work, are presented in Table S6.

### 4.2. Identification and Chromosomal Localization of bZIP Gene Family Members in P. frutescens

The genome annotation data for *P. frutescens* were obtained from the *Perilla frutescens* var. *frutescens* genome database (https://www.ncbi.nlm.nih.gov/genome/?term=txid48386 (accessed on 9 March 2023)). AtbZIP sequences from *Arabidopsis* were acquired from the *Arabidopsis* Information Resource (TAIR) website (https://www.arabidopsis.org/index.jsp (accessed on 9 March 2023)). Then, all the candidate PfbZIPs were identified by the online tools NCBI CD-Search (https://www.ncbi.nlm.nih.gov/Structure/cdd/wrpsb.cgi (accessed on 10 March 2023)) and SMART database (http://smart.embl-heidelberg.de/ (accessed on 10 March 2023)). ProtParam from ExPasy online tools was utilized to analyze the physicochemical properties of the identified PfbZIP members. The chromosomal locations of all the PfbZIP genes were obtained from the genome annotation data of *P. frutescens*. MapChart 2.32 was subsequently used to map the physical location of PfbZIP genes on the chromosome from the short-arm telomeres to the long-arm telomeres [6,55].

### 4.3. Multiple Sequence Alignment and Construction of a Phylogenetic Tree

Multiple sequence alignments of the PfbZIP proteins were conducted using Jalview 2.11.2.7 software. According to the alignment of the bZIP conserved domains between PfbZIPs and AtbZIPs, the neighbor-joining method (N-J) was employed to establish a phylogenetic tree utilizing MEGA 11.0.9 software, and the bootstrap test was set to 1000 times [6,12]. Finally, all the confirmed PfbZIP members were divided into different groups based on the grouping of AtbZIP members.

### 4.4. Analysis of Gene Duplication Events

Segmental duplication analysis of the *P. frutescens* genome was conducted by MCScanX (Multiple Collinearity Scan Tool Kit) in TBtools 2.0, and the entire analytic process was executed with default settings. The tandem duplication event is defined as the occurrence of two or more genes within 200 kb on the same chromosome [60]. The synteny relationships between the genomes of *P. frutescens* and two distinct species, *A. thaliana* and *S. indicum*, were identified and analyzed. The *S. indicum* data was obtained from the NCBI GenBank database (https://www.ncbi.nlm.nih.gov/genome/11560?genome_assembly_id=49670 (accessed on 13 March 2023)).

### 4.5. GO Annotation and KEGG Enrichment Analysis

To confirm the Gene Ontology (GO, http://www.geneontology.org/ (accessed on 14 March 2023)) terms among all the *PfbZIP* genes in the database, we mapped each term and subsequently calculated the number of *PfbZIP* genes linked to each GO term. A list of genetic variations was generated based on their functional annotation in the GO database, and the numbers of *PfbZIP* genes were tallied separately. The hypergeometric test was applied to identify significantly enriched GO terms (FDR < 0.05) among *PfbZIP* gene members in comparison with the background, thereby determining the major functions of different *PfbZIP* genes.

The Kyoto Encyclopedia of Genes and Genomes (KEGG, http://www.kegg.jp/ (accessed on 14 March 2023)) database facilitates the comprehensive exploration of gene functions, genomic interactions, and functional annotations. Genes can be classified by the pathways or by the functions they are involved in using the KEGG database. The hypergeometric test was applied to identify significantly enriched pathways (FDR < 0.05) among *PfbZIP* gene members in comparison with the background, thereby determining the major metabolic and signal transduction pathways in which different *PfbZIP* genes are involved.

### 4.6. Expression Profiling of PfbZIP Genes Based on RNA-Seq and RT-qPCR

To investigate the expression profiles of all the *PfbZIP* genes in developing perilla seeds, we obtained the transcriptome profiles of *PfbZIP* genes from the internal transcriptome sequencing database in our lab. Subsequently, we extracted the FPKM value (number of fragments per kilobase exon per million fragments), and then a heat map illustrating their expression patterns was generated using TBtools 2.0.

Total RNA from perilla-developing seeds was extracted utilizing the EASY spin Plus Plant RNA kit (Aidlab Biotechnologies Co., Ltd., Beijing, China). The first-strand cDNAs were generated using the StarScript II RT Mix with gDNA (GenStar BioSolutions, Beijing, China) following the manufacturer’s instructions. Primer sequences were provided in Table S7. RT-qPCR amplification reactions were conducted via the TB Green^®^ Premix Ex Taq™ II (Tli RNaseH Plus) (TaKaRa, Beijing, China). The experiments were conducted on a CFX96 PCR system (Bio-Rad, Hercules, CA, USA). The *PfActin* gene was used as the internal reference gene [6]. The test was performed using three independent biological samples with three technical replicates. Finally, the relative expression level was calculated by the 2^−ΔΔCt^ method [70].

### 4.7. Prediction of Target Genes Regulated by the bZIP Transcription Factor

To explore the regulatory role of PfbZIP TFs on the expressions of genes related to lipid/oil metabolism, a set of oil-related genes was selected for promoter *cis*-element analysis. The 2000 bp promoter regions upstream for these selected genes were obtained through TBtools 2.0, and then cis-acting element examination was conducted using the PlantCARE database (https://bioinformatics.psb.ugent.be/webtools/plantcare/html/ (accessed on 20 March 2023)) [12]. The identification of target genes was conducted by exploiting the specific recognition and binding capability of bZIP TFs to the A/C/T/CACGTG/C/T/A or GCCACGTGGA motif sequences [22,25]. We further analyzed the expression correlation between these oil-related genes and PfbZIP TFs using their FPKM values.

### 4.8. Gene Cloning and Plasmid Construction

Gene-specific primers (primer sequences provided in Table S7) were designed according to the CDS sequences of the *PfbZIP52* and *PfbZIP85* genes. The TransStart^®^ FastPfu Fly DNA Polymerase (TransGen Biotech, Beijing, China) was utilized for the RT-PCR amplification reactions. The RT-PCR amplicons of *PfbZIP52* and *PfbZIP85* genes were ligated into the pGADT7 prey vector (Figure S3) utilizing the ClonExpress Ultra One Step Cloning Kit (Vazyme, Nanjing, China) through the one-step cloning method. Subsequently, these constructs were transformed into competent cells of *Escherichia coli* DH5α [6]. The positive clones were identified via PCR and gene sequencing alignment (Tsingke Biotechnology Co., Ltd., Xi’an, China).

The specific sequences of the promoter region of *PfGPAT1* and *PfLPAT1B* genes containing ACGT elements were synthesized by Nanjing Ruiyuan Biotechnology Co., Ltd. (Nanjing, China), and the synthesized sequences were separately ligated into the pHIS2 vector to construct bait vectors pHIS2-proPfGPAT1 and pHIS2-proPfLPAT1B, respectively (Figure S3).

Similarly, *PfbZIP85* CDS was cloned and then individually inserted into three expression vectors, including the expression vector pCAMBIA1300/GFP carrying the GFP signal protein, the constitutive plant overexpression vector pCAMBIA1303, and the seed-specific overexpression vector pJC-Gly-DSRB using one-step cloning (the primer sequences are shown in Table S7) to form the expression vectors pCAMBIA1300-*PfbZIP85*/GFP, pCAMBIA1303-*PfbZIP85*, and pJC-Gly-DSRB-*PfbZIP85* (Figure S3), respectively.

The promoter sequence of the *PfLPAT1B* gene was cloned from perilla by RT-PCR using total DNA and subsequently inserted into the expression vector pCAMBIA1301 to obtain a recombinant plasmid pCAMBIA1301-pro*PfLPAT1B* (Figure S3).

### 4.9. The Yeast One-Hybrid (Y1H) Assay

The yeast one-hybrid (Y1H) assay was performed following the procedures described by Clontech (Takara). Bait vectors (pHIS2-proPfGPAT1 or pHIS2-proPfLPAT1B) and the AD plasmid (pGADT7) were transformed into Y187 yeast strain competent cells and cultured on SD-TL (Coolaber Technology Company, Beijing, China) medium at 30 °C for 3 d. pHIS2-P53 and pGAD53m were co-transfected into the yeast cell as the positive controls. Similarly, pHIS2-proPfGPAT1 + pGADT7-PfbZIP52 and pHIS2-proPfLPAT1B + pGADT7-PfbZIP85 were co-transformed into the yeast cell as the experimental group, respectively.

The single bacterial colony for pHIS2-proPfGPAT1 + pGADT7 or pHIS2-proPfLPAT1B + pGADT7 and pHIS2-P53 + pGAD53m were randomly selected from each transformation reaction plate and then cultured on SD-TLH with or without 3-amino-1,2,4-triazole (0, 10, 20, 30, 40, 50, 75, 100 mmol/L 3AT) at 30 °C for 3 d.

The concentration of 3AT for screening the positive colonies was determined by the self-activation detection concentration of 3AT of the pHIS2-proPfGPAT1 and pHIS2-proPfLPAT1B bait vectors. Each sample of pHIS2-proPfGPAT1 + pGADT7-PfbZIP52 or pHIS2-proPfLPAT1B + pGADT7-PfbZIP85 was randomly selected and then spotted on SD-TL and SD-TLH with or without an appropriate concentration of 3AT medium for 3 d to 5 d cultivation.

### 4.10. Subcellular Localization of PfbZIP85 Protein and GUS Activity Assay

To investigate the subcellular localization of PfbZIP85 protein, the recombinant fusion plasmid 35S::PfbZIP85::GFP (pCAMBIA1300-PfbZIP85/GFP) and the control plasmid 35S::GFP were transiently expressed in *N. benthamiana* leaf epidermal cells through Agrobacterium-mediated transformation [6]. After the tobacco plants were grown at 25 °C for more than 36 h, the transfected tobacco leaf samples were treated with DAPI reagent (Solarbio Life Sciences, Beijing, China), followed by observation of fluorescence signals utilizing a laser scanning confocal microscope (Leica TCS SP8).

### 4.11. GUS Stain and GUS Activity Assays

*Agrobacterium* GV3101 strain harboring 35S::PfbZIP85 and pro*PfLPAT1B*::GUS (pCAMBIA1301-pro*PfLPAT1B*) plasmids were transiently co-infiltrated into tobacco leaves. At the same time, the pro*PfLPAT1B*::GUS plasmid alone was infiltrated into tobacco leaves as the control. After 48 h of culturing, the infiltrated leaves were subjected to GUS staining with a kit (Beijing Huayueyang Science and Technology Co., Ltd., Beijing, China), followed by observation under a stereoscopic microscope. Moreover, an RT-qRCR assay was employed to assess the relative expression level of the *GUS* gene.

### 4.12. Heterologous Overexpression of PfbZIP85 in Tobacco Plant

The recombinant plant overexpression vector pCAMBIA1303-PfbZIP85 and the seed-specific vector pJC-Gly-DSRB-PfbZIP85 were genetically transformed in tobacco by the *Agrobacterium*-mediated method, respectively. The leaves of aseptic tobacco seedlings were transformed via *Agrobacterium* infiltration [71]. And then, the positive transgenic tobacco plants were identified using RT-PCR (primer sequences seen in Table S7). Subsequently, the positive transgenic tobacco plants were selected for phenotypic assessment.

### 4.13. Lipid Extraction and Fatty Acid Analysis

Total lipids were extracted from seeds or leaf tissues using chloroform–methanol–KCl (2:1:1, *v*/*v*/*v*) according to the method of Vanhercke et al. [13]. The specific operation is carried out according to the following steps: Freeze-dried perilla seeds, tobacco seeds, and leaf tissues were transferred to a 10 mL stopper glass test tube containing a chloroform–methanol mixture and mixed with shaking on a vortex mixer for 10 min, and then 0.1 M KCl was supplemented and mixed for a further 5 min. Finally, the mixture was centrifuged at 4000 rpm for 10 min to collect the lower lipid phase. The remaining phase was washed once with chloroform, and the lipid phase was subsequently combined with the earlier extract. The collected chloroform was completely evaporated to dryness with nitrogen gas (N_2_) and stored at −20 °C until further use.

Neutral lipid classes (TAG) were separated from total lipids by thin-layer chromatography (TLC) using a silica gel plate (100 mm × 200 mm, 0.20–0.25 mm thickness) with the developing agent of hexane–methyl tert-butyl ether–acetic acid (80:20:2, *v*/*v*/*v*), and then visualized by staining with iodine vapor. The TAG strip on the silicone plate was carefully scraped off using a blade and transferred into a stopper glass test tube, and then the fatty acid methyl ester (FAME) was prepared following the modified methods by Li et al. [72]. 0.5 mL of sodium methanolic solution (containing 0.001% BHT) was added to the aforementioned glass test tube, followed by the addition of tri-heptadecanoin (tri-17:0) (10 mg/mL in chloroform) as an internal standard. The test tube was sealed and incubated at 80 °C for 2 h. After the test tube containing the mixture was cooled, 0.5 mL of hexane (containing 0.001% BHT) and 1 mL of 0.9% KCl solution were added to each test tube and then mixed well. The mixture was centrifuged at 4000 rpm for 8 min. The top layer was collected and then completely evaporated to dryness with N_2_, followed by the addition of 0.2 mL of hexane to dissolve the extract and transferring into GC auto-sampler vials.

FAMEs were detected and analyzed utilizing an Agilent 7890B gas chromatograph (GC) equipped with a hydrogen flame detector and a G3903-63011 column (30 m × 0.25 mm × 0.25 µm). The initial temperature was 50 °C for 0.5 min, then to 194 °C at 30 °C/min, holding for 3.5 min, and then to 240 °C at 5 °C/min, holding for one min. The temperature of the FID detector was set at 280 °C, with hydrogen flowing at a rate of 40 mL/min, air at 400 mL/min, and nitrogen at 25 mL/min. The evaporator temperature was set at 250 °C. The split flow was not used, and the injection volume was 1 µL. The areas for each fatty acid, including the tri-17:0 standard, were collected and subsequently used to calculate the TAG content (µg/mg or µg/seed) and each fatty acid level (% of TAG).

### 4.14. Analysis of Other Agronomy Traits in Transgenic Tobacco Lines

The starch, protein, and soluble sugar contents of *PfbZIP85*-expressing tobacco plants were determined using assay kits specific for starch, protein, and soluble sugar content (Beijing Solarbio Science and Technology Co., Ltd., Beijing, China), respectively. In addition, the leaf dry weight, photosynthetic rate (Pn), thousand-seed weight, and germination rate of *PfbZIP85*-expressing plants were detected quantitatively.

### 4.15. Data Analysis

All samples were tested in triplicate, at least. The software DPS (new version 9.01) was used to statistically analyze the data with the single-factor multiple comparison method. Values are presented as mean ± SD (*n* = 3). Different lowercase letters indicate significant differences between treatments at *p* < 0.05.

## 5. Conclusions

In the present study, 101 PfbZIP TF members were first identified in the *P. frutescens* genome, being classified into eleven different groups (Group A, B, C, D, E, F, G, H, I, S, and UC) based on phylogenetic, gene structure, and protein conserved motif analysis. The diverse expression patterns were detected for different *PfbZIP* genes during perilla seed development. Particularly, *PfbZIP52* expression exhibited a positive correlation with that of the *PfGPAT1* gene, whereas the expression of *PfbZIP85* displayed a significantly negative correlation with that of the *PfLPAT1B* gene. The Y1H assay and GUS activity test revealed that PfbZIP85 TF negatively regulated *PfLPAT1B* expression through physical interaction with the promoter of the *PfLPAT1B* gene. Functional characterization of PfbZIP85 in heterogeneous tobacco plants evidenced that PfbZIP85 mediated regulation of FA/oil biosynthesis and accumulation by modulating expressions of the lipid-related enzyme genes. These findings provide a valuable foundation for further functional examination of PfbZIP members, advancing our understanding of the PfbZIP-mediated regulation network for the biosynthesis of vegetable oils enriched with target FAs.

## Figures and Tables

**Table 1:** Prediction of *cis*-elements in promoter regions of genes related to FA biosynthesis in perilla.

Gene Name		Gene ID	Binding Sequence
*PfKAS2*	β-Ketoacyl-acp synthase 2	C2S51_000860	CACGTA
*PfSAD2*	Δ9-Stearoyl-ACP desaturase 2	C2S51_009736	TACGTG
*PfFAD6*	ω-6 fatty acid desaturase 6	C2S51_002817	GACGTC/TACGTA
*PfGPAT1*	Glycerol-3-Phosphate Acyltransferase 1	C2S51_037878	AACGTG/CAACGTTA
*PfLPAT1B*	lysophosphatidic acid acyltransferase 1B	C2S51_002830	TACGTG/AACGTG/AACGTT
*PfDGAT2A*	Diacylglycerol Acyltransferase 2A	C2S51_029109	CACGTG/TACGTG
*PfDGAT3A*	Diacylglycerol Acyltransferase 3A	C2S51_015166	CACGTT/CACGTC/TACGTG
*PfPDAT2B*	Phospholipid Diacylglycerol Acyltransferase	C2S51_011488	CACGTG/TACGTG

## Data Availability

Data supporting the discovery of our work are available within this paper and its Supplementary Materials.

## Supplementary Materials

The following supporting information can be downloaded at: https://www.mdpi.com/article/10.3390/ijms25084375/s1.
