# Supplementary material for: PfbZIP85 Transcription Factor Mediates ω-3 Fatty Acid-Enriched Oil Biosynthesis by Down-Regulating PfLPAT1B Gene Expression in Plant Tissues
Source: Int J Mol Sci. 2024 Apr 16;25(8):4375. doi: 10.3390/ijms25084375 (PMC11050522; doi:10.3390/ijms25084375)
Supplement: Supplementary file 1 [file ijms-25-04375-s001.zip › ijms-2936241-supplementary.pdf]

PfbZIP85 transcription factor mediates ω-3 fatty acid-enriched oil biosynthesis by down-regulating *PfLPAT1B* gene expression in plant tissues

Additional file

Figure S1. Chromosomal locations of *PfbZIP* genes.

Figure S2. PCR cloning of *PfbZIP52* and *PfbZIP85* genes ORF.

Figure S3. Diagrams showing different expression vectors.

Figure S4. Detection of self-activation of pHIS2-proPfGPAT1 and pHIS2-proPfLPAT1B bait vectors, respectively.

Figure S5. RT-PCR cloning of *PfLPAT1B* promoter.

Figure S6. PCR detection of transgenic tobacco plant overexpressing of *PfGPAT85* gene.

Table S1. The number of bZIP genes in the reported species.

Table S2. Basic physicochemical properties of PfbZIP members.

Table S3. Duplication gene pairs of *PfbZIP* genes.

Table S4. Duplication gene pairs of *bZIP* genes from *P. frucescens* and *A. thaliana.*

Table S5. Duplication gene pairs of *bZIP* genes from *P. frucescens* and *S. indicum.*

Table S6. Bacterial and yeast strains and plasmids used in this study.

Table S7. Primer sequences used in this study.

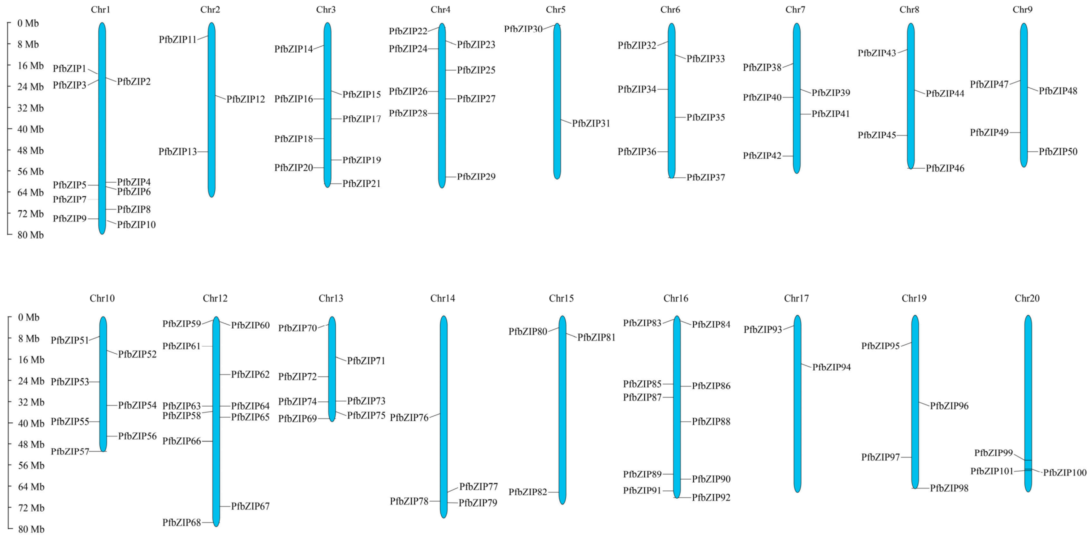

Figure S1. Chromosomal locations of *PfbZIP* genes. The relative length of the chromosomes is millions of base pairs (Mb).

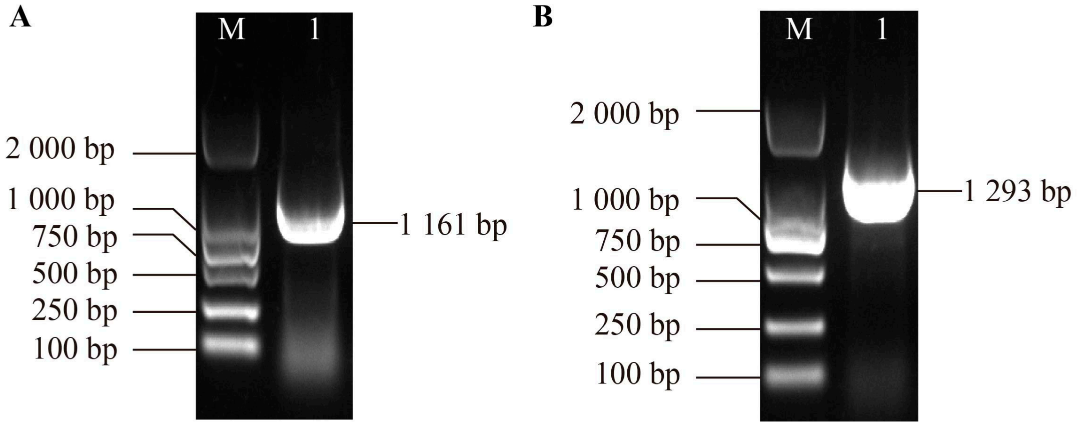

Figure S2. PCR cloning of *PfbZIP52* **(A)** and *PfbZIP85* **(B)** genes ORF. M, DNA Marker DL 2000; 1, The PCR product of full-length ORF of *PfbZIP52* **(A)** or *PfbZIP85* **(B)** gene.

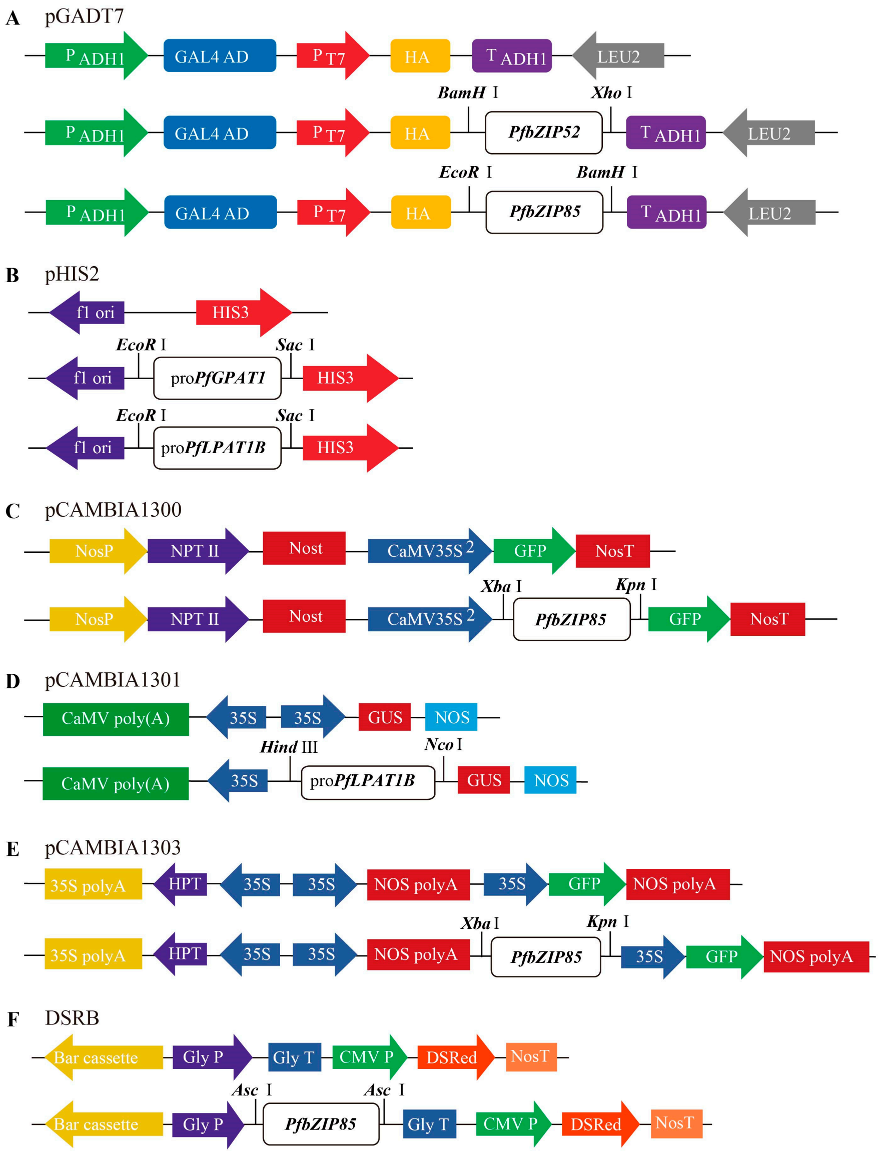

Figure S3. Diagrams showing different expression vectors. **(A)** Diagrams showing pGADT7+*PfbZIP52* and pGADT7+*PfbZIP85* prey vectors. **(B)** Diagrams showing pHIS2+*proPfGPAT1* and pHIS2+*proPfLPAT1B* bait vectors. **(C)** Diagram showing pCAMBIA1300+*PfbZIP85*/GFP expression vector. **(D)** Diagram showing pCAMBIA1301+*proPfLPAT1B* GUS expression vector. **(E)** Diagram showing pCAMBIA1303+*PfbZIP85* constitutive plant expression vector. **(F)** Diagram showing pJC-Gly-DSRB+*PfbZIP85* seed-specific plant expression vector.

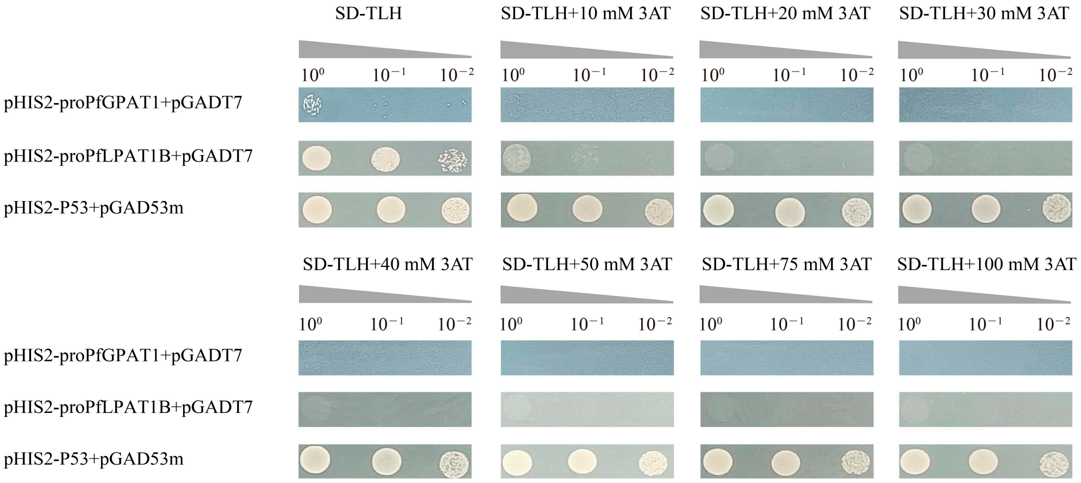

Figure S4. Detection of self-activation of pHIS2-proPfGPAT1 and pHIS2-proPfLPAT1B bait vectors, respectively.

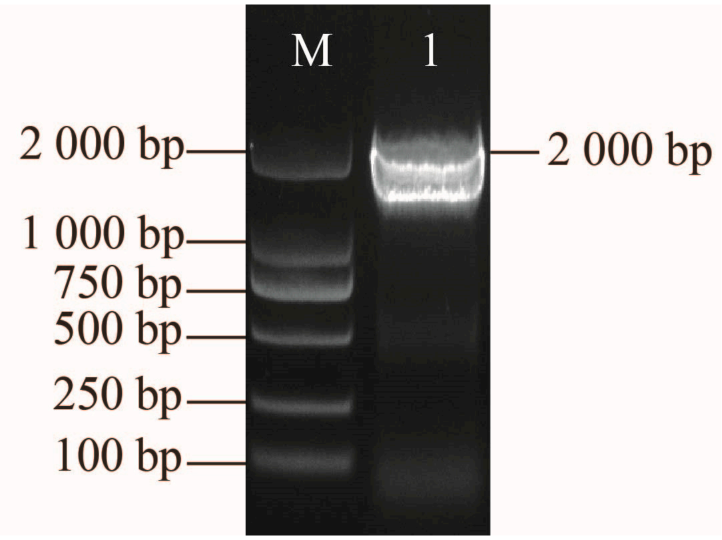

Figure S5. RT-PCR cloning of *PfLPAT1B* promoter. M, DNA Marker DL 2000; 1, The PCR product of the 2000 bp promoter region of *PfLPAT1B* gene.

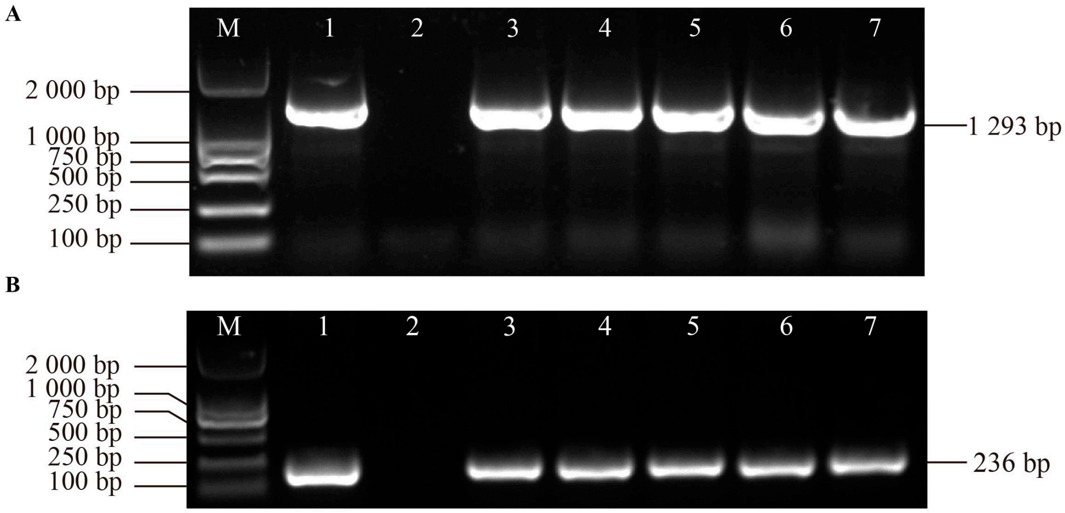

Figure S5. PCR detection of transgenic tobacco plant overexpressing of *PfGPAT85* gene. PCR detection of genomic level **(A)** and transcription level **(B)** of *PfGPAT85*-transgenic tobacco lines. M, DNA Marker DL 2000; 1, pCAMBIA1303+*PfbZIP85* recombinant plasmid; 2, the wild-type (WT) tobacco plant lines; 3–7, *PfbZIP85*-expressing tobacco plant lines.

Table S1. The number of bZIP genes in the reported species.

Species	Number	References
Ricinus communis	100	Jin Z, Xu W, Liu A. Genomic surveys and expression analysis of bZIP gene family in castor bean (Ricinus communis L.). *Planta*. 2014;**239**(2):299-312.
Arabidopsis thaliana	75	Jakoby M, Weisshaar B, Dröge-Laser W *et al.* bZIP Transcription Factors in Arabidopsis. *Trends Plant Sci.* 2002;**7**(3):106-111.
Oryza sativa	89	Nijhawan A, Jain M, Tyagi AK *et al.* Genomic survey and gene expression analysis of the basic leucine zipper transcription factor family in rice. *Plant Physiol.* 2008;**146**(2):333-350.
Zea mays	125	Wei K, Chen J, Wang Y *et al.* Genome-wide analysis of bZIP-encoding genes in maize. *DNA Res.* 2012;**19**(6):463-476.
Brassica napus	247	Zhou Y, Xu D, Jia L *et al.* Genome-Wide Identification and Structural Analysis of bZIP Transcription Factor Genes in Brassica napus. *Genes (Basel)*. 2017;**8**(10):288.
Glycine max	160	Zhang M, Liu Y, Shi H *et al.* Evolutionary and expression analyses of soybean basic Leucine zipper transcription factor family. *BMC Genomics*. 2018;**19**(1):159.
Sesamum indicum	63	Wang Y, Zhang Y, Zhou R *et al.* Identification and characterization of the bZIP transcription factor family and its expression in response to abiotic stresses in sesame. *PLoS One*. 2018;**13**(7):e0200850.
Arachis duranensis	50	Wang Z, Yan L, Wan L *et al.* Genome-wide systematic characterization of bZIP transcription factors and their expression profiles during seed development and in response to salt stress in peanut. *BMC Genomics*. 2019;**20**(1):51.

Table S2. Basic physicochemical properties of PfbZIP members.

Name	Gene ID	Chr	Start	End	Protein length	MW (kDa)	pI	Group
PfbZIP1	C2S51_001169	1	18793982	18795155	319	35.16	7.83	A
PfbZIP2	C2S51_001228	1	19969850	19972784	779	84.22	5.62	B
PfbZIP3	C2S51_001269	1	20645497	20648451	435	48.29	6.53	D
PfbZIP4	C2S51_002235	1	57885237	57886406	319	35.11	7.83	A
PfbZIP5	C2S51_002276	1	58894137	58897059	776	83.68	5.57	B
PfbZIP6	C2S51_002305	1	59501620	59504560	435	48.31	6.78	D
PfbZIP7	C2S51_002523	1	63876300	63878590	436	46.76	8.95	A
PfbZIP8	C2S51_002704	1	67469834	67473896	450	47.7	6.65	C
PfbZIP9	C2S51_002908	1	70879017	70883585	650	71.83	6.48	A
PfbZIP10	C2S51_002937	1	71399582	71402681	430	47.88	7.82	D
PfbZIP11	C2S51_019884	2	4802218	4805547	470	51.71	6.33	D
PfbZIP12	C2S51_020224	2	26452169	26457035	328	36.73	7.89	D
PfbZIP13	C2S51_020868	2	46646977	46650157	401	43.84	6.47	I
PfbZIP14	C2S51_021918	3	8246510	8265677	341	39.47	4.84	UC
PfbZIP15	C2S51_022229	3	24577432	24580467	402	43.98	5.8	G
PfbZIP16	C2S51_022321	3	27558449	27561280	558	61.38	6.27	I
PfbZIP17	C2S51_022630	3	34700288	34705109	326	36.36	8.64	D
PfbZIP18	C2S51_022920	3	41990406	41990822	138	15.6	6.74	S
PfbZIP19	C2S51_023242	3	49739451	49740360	198	22.81	9.9	UC
PfbZIP20	C2S51_023394	3	52355613	52356329	238	26.3	5.82	F
PfbZIP21	C2S51_023820	3	58471737	58472417	226	25.28	9.69	A
PfbZIP22	C2S51_024072	4	1628422	1629564	277	31.34	7.22	UC
PfbZIP23	C2S51_024442	4	6525446	6526162	238	26.46	5.94	F
PfbZIP24	C2S51_024604	4	9111060	9112074	198	22.86	10.1	UC
PfbZIP25	C2S51_024987	4	16785430	16785846	138	15.6	6.74	S
PfbZIP26	C2S51_025355	4	24517366	24522125	326	36.36	8.64	D
PfbZIP27	C2S51_025469	4	27273165	27274419	282	29.61	5.41	A
PfbZIP28	C2S51_025706	4	32607278	32610149	562	62.02	6.12	I
PfbZIP29	C2S51_026235	4	55653134	55657225	382	42.31	6.03	D
PfbZIP30	C2S51_026407	5	759290	759757	155	18.47	8.66	C
PfbZIP31	C2S51_027522	5	34787058	34791922	328	36.74	7.89	D
PfbZIP32	C2S51_028307	6	6636665	6637258	197	22.8	5.97	S

PfbZIP33	C2S51_028562	6	11512935	11514484	385	42.86	8.56	A
PfbZIP34	C2S51_029023	6	23898663	23905096	358	37.89	5.65	G
PfbZIP35	C2S51_029304	6	34081401	34084011	361	40.59	6.73	D
PfbZIP36	C2S51_029471	6	46323536	46326583	762	81.93	6.36	B
PfbZIP37	C2S51_029630	6	55769789	55770205	138	15.58	6.74	S
PfbZIP38	C2S51_029973	7	14672249	14673613	225	26.05	9.37	UC
PfbZIP39	C2S51_030271	7	23905348	23905875	175	20.32	5.98	S
PfbZIP40	C2S51_030360	7	26864674	26867886	337	37.28	5.6	I
PfbZIP41	C2S51_030554	7	32890032	32893589	311	35.26	6.52	UC
PfbZIP42	C2S51_031284	7	47909162	47909719	185	21.58	5.44	S
PfbZIP43	C2S51_032111	8	9548738	9550409	366	41.31	6.62	D
PfbZIP44	C2S51_032456	8	24101605	24105937	364	39.68	7.93	C
PfbZIP45	C2S51_032655	8	40567692	40571478	395	41.69	6.68	G
PfbZIP46	C2S51_033010	8	52369743	52370727	112	12.07	10.88	H
PfbZIP47	C2S51_033598	9	20730351	20730878	175	20.23	5.98	S
PfbZIP48	C2S51_033661	9	23164144	23167366	337	37.21	5.51	I
PfbZIP49	C2S51_034184	9	39346628	39350425	415	46.11	7.13	UC
PfbZIP50	C2S51_034508	9	46238775	46239332	185	21.5	5.45	S
PfbZIP51	C2S51_035146	10	7116760	7117353	197	22.81	5.97	S
PfbZIP52	C2S51_035357	10	12200992	12202559	386	42.9	7.76	A
PfbZIP53	C2S51_035745	10	23570435	23577057	358	37.89	5.65	G
PfbZIP54	C2S51_035968	10	32073021	32075623	361	40.62	6.73	D
PfbZIP55	C2S51_036057	10	37770900	37771610	236	27.35	10.55	S
PfbZIP56	C2S51_036102	10	43273947	43277007	764	82.16	6.28	B
PfbZIP57	C2S51_036246	10	48798773	48799189	138	15.57	6.74	S
PfbZIP58	C2S51_000027	12	34366593	34369459	385	41.39	6.15	G
PfbZIP59	C2S51_003355	12	1273545	1275527	446	48.86	7.24	I
PfbZIP60	C2S51_003384	12	1905606	1905989	127	14.25	6.72	S
PfbZIP61	C2S51_003549	12	10807494	10807850	118	14	9.57	S
PfbZIP62	C2S51_003720	12	21057855	21059759	283	31.54	6.67	E
PfbZIP63	C2S51_004167	12	32285335	32287342	261	28.53	8.86	I
PfbZIP64	C2S51_004172	12	32349964	32351611	175	20.05	9.51	A
PfbZIP65	C2S51_004384	12	36438077	36438532	151	16.83	7.87	S
PfbZIP66	C2S51_004789	12	45071361	45077293	486	54.08	6.93	D
PfbZIP67	C2S51_005961	12	68525641	68529725	322	35.83	5.36	C
PfbZIP68	C2S51_006383	12	74467200	74468343	277	31.4	6.87	UC

PfbZIP69	C2S51_000426	13	36799720	36802620	385	41.34	6.15	G
PfbZIP70	C2S51_037514	13	2858269	2858691	140	16.47	6.71	S
PfbZIP71	C2S51_037725	13	14647979	14649837	283	31.54	7.07	E
PfbZIP72	C2S51_037958	13	21785305	21787653	310	34.78	6.1	E
PfbZIP73	C2S51_038307	13	30555986	30557988	260	28.37	8.86	I
PfbZIP74	C2S51_038314	13	30633998	30635637	175	20.22	9.21	A
PfbZIP75	C2S51_038468	13	34416257	34416712	151	16.78	6.96	S
PfbZIP76	C2S51_007199	14	35387002	35388358	226	26.18	8.88	UC
PfbZIP77	C2S51_007705	14	63832104	63833316	206	23.84	5.37	A
PfbZIP78	C2S51_007809	14	67133077	67134094	279	30.15	5.01	A
PfbZIP79	C2S51_007829	14	67702351	67704714	174	19.37	9.98	I
PfbZIP80	C2S51_008317	15	4468205	4470711	567	62.01	6.21	I
PfbZIP81	C2S51_008371	15	6453444	6454382	312	34.46	5.85	F
PfbZIP82	C2S51_009704	15	63666023	63667961	340	38.02	6.56	I
PfbZIP83	C2S51_010049	16	1228350	1236456	866	95.6	9.11	I
PfbZIP84	C2S51_010072	16	1719047	1719421	124	13.95	6.72	S
PfbZIP85	C2S51_010603	16	24681391	24684518	430	47.91	6.74	D
PfbZIP86	C2S51_010640	16	25245422	25250053	656	72.38	6.59	A
PfbZIP87	C2S51_010886	16	29375877	29379936	449	47.57	6.65	C
PfbZIP88	C2S51_011446	16	38092456	38098382	487	54.26	6.95	D
PfbZIP89	C2S51_012564	16	57300436	57310271	1269	142.58	8.15	H
PfbZIP90	C2S51_012715	16	59036229	59040312	322	35.85	5.36	C
PfbZIP91	C2S51_013043	16	63122028	63122594	188	22.16	5.44	S
PfbZIP92	C2S51_013256	16	65779078	65779758	226	25.23	9.69	A
PfbZIP93	C2S51_013540	17	3909599	3912101	342	37.98	6.42	I
PfbZIP94	C2S51_013941	17	17453382	17455851	565	61.79	6.21	I
PfbZIP95	C2S51_017148	19	9760251	9761921	366	41.31	7.02	D
PfbZIP96	C2S51_017863	19	31255368	31259139	395	41.71	6.68	G
PfbZIP97	C2S51_018122	19	51417815	51422060	364	39.72	8.57	C
PfbZIP98	C2S51_018358	19	62376272	62377284	112	12.1	10.65	H
PfbZIP99	C2S51_019255	20	52499069	52500317	206	23.98	5.37	A
PfbZIP100	C2S51_019333	20	55491444	55492449	277	29.91	5.11	A
PfbZIP101	C2S51_019353	20	56052495	56055240	305	33.7	9.37	I

Table S3. Duplication gene pairs of *PfbZIP* genes.

ID		ID	
C2S51_001169	C2S51_002235	C2S51_030271	C2S51_033598
C2S51_001169	C2S51_010640	C2S51_030271	C2S51_035146
C2S51_001169	C2S51_002908	C2S51_002235	C2S51_010640
C2S51_002937	C2S51_010603	C2S51_002235	C2S51_002908
C2S51_020224	C2S51_022630	C2S51_030360	C2S51_033661
C2S51_020224	C2S51_025355	C2S51_031284	C2S51_034508
C2S51_020224	C2S51_027522	C2S51_031284	C2S51_013043
C2S51_022229	C2S51_000027	C2S51_032111	C2S51_035968
C2S51_022229	C2S51_000426	C2S51_032111	C2S51_017148
C2S51_022321	C2S51_025706	C2S51_032456	C2S51_018122
C2S51_022321	C2S51_008317	C2S51_032655	C2S51_017863
C2S51_022321	C2S51_013941	C2S51_033010	C2S51_018358
C2S51_022630	C2S51_025355	C2S51_033598	C2S51_035146
C2S51_022630	C2S51_027522	C2S51_033598	C2S51_013043
C2S51_022920	C2S51_024987	C2S51_002276	C2S51_029471
C2S51_022920	C2S51_026407	C2S51_034508	C2S51_013043
C2S51_022920	C2S51_029630	C2S51_035968	C2S51_017148
C2S51_022920	C2S51_036246	C2S51_000027	C2S51_000426
C2S51_023242	C2S51_024604	C2S51_003355	C2S51_010049
C2S51_001228	C2S51_029471	C2S51_002305	C2S51_002937
C2S51_001228	C2S51_002276	C2S51_002305	C2S51_010603
C2S51_023394	C2S51_024442	C2S51_003384	C2S51_010072
C2S51_023820	C2S51_013256	C2S51_003549	C2S51_037514
C2S51_024072	C2S51_006383	C2S51_003720	C2S51_037725
C2S51_024987	C2S51_029630	C2S51_004167	C2S51_019353
C2S51_024987	C2S51_036246	C2S51_004167	C2S51_038307
C2S51_025355	C2S51_027522	C2S51_004167	C2S51_007829
C2S51_025706	C2S51_008317	C2S51_004172	C2S51_038314
C2S51_025706	C2S51_013941	C2S51_004172	C2S51_007809
C2S51_001269	C2S51_002937	C2S51_004384	C2S51_038468
C2S51_001269	C2S51_002305	C2S51_004789	C2S51_011446
C2S51_001269	C2S51_010603	C2S51_005961	C2S51_012715
C2S51_028307	C2S51_030271	C2S51_038307	C2S51_019353

C2S51_028307	C2S51_033598	C2S51_038307	C2S51_007829
C2S51_028307	C2S51_035146	C2S51_007705	C2S51_019255
C2S51_028562	C2S51_035357	C2S51_007809	C2S51_019333
C2S51_029023	C2S51_035745	C2S51_007829	C2S51_019353
C2S51_029304	C2S51_032111	C2S51_002704	C2S51_010886
C2S51_029304	C2S51_035968	C2S51_008317	C2S51_013941
C2S51_029304	C2S51_017148	C2S51_009704	C2S51_013540
C2S51_029471	C2S51_036102	C2S51_002908	C2S51_010640
C2S51_029630	C2S51_036246		

Table S4. Duplication gene pairs of *bZIP* genes from *P. frucescens* and *A. thaliana*.

P. frutescens	*A. thaliana*	*P. frutescens*	*A. thaliana*
C2S51_001228	NM_129659.3	C2S51_023394	NM_115055.3
C2S51_001228	NM_115525.2	C2S51_024442	NM_115055.3
C2S51_002276	NM_129659.3	C2S51_024987	NM_116107.2
C2S51_002276	NM_115525.2	C2S51_028307	NM_101230.4
C2S51_002523	NM_001198254.2	C2S51_028307	NM_126441.1
C2S51_002523	NM_001203005.2	C2S51_028562	NM_001336591.1
C2S51_002523	NM_001342246.1	C2S51_029023	NM_119837.4
C2S51_002908	NM_001336890.1	C2S51_029304	NM_106441.4
C2S51_003384	NM_106193.4	C2S51_029304	NM_180942.3
C2S51_000027	NM_130190.3	C2S51_029304	NM_121041.5
C2S51_000027	NM_116342.3	C2S51_029471	NM_115525.2
C2S51_000027	NM_106193.4	C2S51_029630	NM_116107.2
C2S51_000027	NM_127373.2	C2S51_029630	NM_124322.3
C2S51_000027	NM_119625.3	C2S51_030271	NM_126441.1
C2S51_004789	NM_001331770.1	C2S51_030360	NM_129624.5
C2S51_005961	NM_122389.4	C2S51_031284	NM_121588.3
C2S51_008317	NM_120050.4	C2S51_031284	NM_123241.3
C2S51_008371	NM_127229.3	C2S51_032111	NM_106441.4
C2S51_008371	NM_119670.5	C2S51_032111	NM_121041.5
C2S51_009704	NM_100488.4	C2S51_032655	NM_102948.4
C2S51_010072	NM_106193.4	C2S51_033598	NM_101230.4
C2S51_010640	NM_001336890.1	C2S51_033598	NM_126441.1
C2S51_011446	NM_001331770.1	C2S51_033661	NM_129624.5
C2S51_012564	NM_001336002.1	C2S51_034508	NM_121588.3
C2S51_012564	NM_119393.3	C2S51_034508	NM_123241.3
C2S51_012715	NM_122389.4	C2S51_035146	NM_101230.4
C2S51_013043	NM_113954.2	C2S51_035146	NM_126441.1
C2S51_013043	NM_121588.3	C2S51_035357	NM_001336591.1
C2S51_013043	NM_123241.3	C2S51_035745	NM_119837.4
C2S51_013941	NM_120050.4	C2S51_035968	NM_106441.4
C2S51_017148	NM_106441.4	C2S51_035968	NM_121041.5
C2S51_017863	NM_102948.4	C2S51_036057	NM_001202651.1
C2S51_019353	NM_103495.4	C2S51_036057	NM_114836.3

C2S51_019884	NM_105536.4	C2S51_036246	NM_116107.2
C2S51_020868	NM_100488.4	C2S51_000426	NM_180038.2
C2S51_022229	NM_130190.3	C2S51_037958	NM_106193.4
C2S51_022229	NM_116342.3	C2S51_037958	NM_127373.2
C2S51_022321	NM_001335735.1	C2S51_037958	NM_119625.3
C2S51_022321	NM_120050.4	C2S51_038468	NM_130190.3
C2S51_022920	NM_116107.2	C2S51_038468	NM_116342.3

Table S5. Duplication gene pairs of *bZIP* genes from *P. frucescens* and *S. indicum*.

P. frutescens	*S. indicum*	*P. frutescens*	*S. indicum*
C2S51_001169	XM_011071683.2	C2S51_022321	XM_011085193.2
C2S51_001169	XM_011080830.2	C2S51_022321	XM_011090244.2
C2S51_001228	XM_020691729.1	C2S51_022630	XM_011077550.2
C2S51_001228	XM_011080786.2	C2S51_022630	XM_011086288.2
C2S51_001269	XM_011077123.2	C2S51_022920	XM_011081252.2
C2S51_001269	XM_011080767.2	C2S51_022920	XM_011084120.2
C2S51_002235	XM_011071683.2	C2S51_023394	XM_020697055.1
C2S51_002235	XM_011080830.2	C2S51_023820	XM_011082182.2
C2S51_002276	XM_020691729.1	C2S51_023820	XM_011091902.2
C2S51_002276	XM_011080786.2	C2S51_024442	XM_020697055.1
C2S51_002305	XM_011077123.2	C2S51_024604	XM_011082424.1
C2S51_002305	XM_011080767.2	C2S51_024987	XM_011081252.2
C2S51_002523	XM_011076125.2	C2S51_024987	XM_011084120.2
C2S51_002523	XM_011089948.2	C2S51_025355	XM_011077550.2
C2S51_002704	XM_020694012.1	C2S51_025355	XM_011086288.2
C2S51_002908	XM_011071683.2	C2S51_025469	XM_011089512.2
C2S51_002908	XM_011080833.2	C2S51_025706	XM_011085193.2
C2S51_002937	XM_011077123.2	C2S51_025706	XM_011090244.2
C2S51_002937	XM_011080767.2	C2S51_026235	XM_011076874.2
C2S51_003355	XM_011076344.2	C2S51_026235	XM_011080551.2
C2S51_003384	XM_011076685.2	C2S51_026407	XM_011081252.2
C2S51_003384	XM_011085319.2	C2S51_026407	XM_011102473.2
C2S51_003549	XM_011075373.2	C2S51_027522	XM_011077550.2
C2S51_003549	XM_020698904.1	C2S51_027522	XM_011086288.2
C2S51_003720	XM_011084427.2	C2S51_028307	XM_011079474.2
C2S51_004167	XM_011103188.2	C2S51_028562	XM_011096053.2
C2S51_004167	XM_011076706.2	C2S51_029023	XM_011098912.2
C2S51_004172	XM_011103049.2	C2S51_029023	XM_011101283.2
C2S51_000027	XM_011095049.2	C2S51_029304	XM_011092274.2
C2S51_004384	XM_011076685.2	C2S51_029304	XM_020698619.1
C2S51_004384	XM_011085319.2	C2S51_029471	XM_020691729.1
C2S51_004789	XM_020696539.1	C2S51_029471	XM_011080786.2
C2S51_005961	XM_011077677.2	C2S51_029630	XM_011084120.2

C2S51_007809	XM_011103049.2	C2S51_029630	XM_011081252.2
C2S51_007829	XM_011103188.2	C2S51_030271	XM_011079474.2
C2S51_008317	XM_011090244.2	C2S51_030360	XM_011078285.2
C2S51_008371	XM_020695942.1	C2S51_030360	XM_011086520.2
C2S51_008371	XM_011094670.2	C2S51_030554	XM_020693145.1
C2S51_009704	XM_020692310.1	C2S51_031284	XM_011087366.2
C2S51_009704	XM_011093336.2	C2S51_031284	XM_011100769.2
C2S51_010049	XM_011076344.2	C2S51_032111	XM_011092274.2
C2S51_010072	XM_011076685.2	C2S51_032111	XM_020698619.1
C2S51_010603	XM_011077123.2	C2S51_032655	XM_011076291.2
C2S51_010603	XM_011080767.2	C2S51_032655	XM_011084006.2
C2S51_010640	XM_011071683.2	C2S51_033010	XM_011083277.2
C2S51_010640	XM_011080830.2	C2S51_033598	XM_011079474.2
C2S51_010886	XM_011093646.2	C2S51_033598	XM_011100769.2
C2S51_011446	XM_020696539.1	C2S51_033598	XM_011078285.2
C2S51_012564	XM_011072866.2	C2S51_033661	XM_011086520.2
C2S51_012564	XM_011083276.2	C2S51_034184	XM_011079579.2
C2S51_012715	XM_011077677.2	C2S51_034184	XM_011096084.2
C2S51_013043	XM_011079474.2	C2S51_034508	XM_011087366.2
C2S51_013043	XM_011087366.2	C2S51_034508	XM_011100769.2
C2S51_013043	XM_011100769.2	C2S51_035146	XM_011079474.2
C2S51_013256	XM_011082182.2	C2S51_035357	XM_011096053.2
C2S51_013256	XM_011091902.2	C2S51_035745	XM_011098912.2
C2S51_013540	XM_020692310.1	C2S51_035745	XM_011101283.2
C2S51_013540	XM_011093336.2	C2S51_035968	XM_011092274.2
C2S51_013941	XM_011090244.2	C2S51_035968	XM_020698619.1
C2S51_017148	XM_011099591.2	C2S51_036057	XM_011075373.2
C2S51_017148	XM_020698619.1	C2S51_036057	XM_020698904.1
C2S51_017863	XM_011076291.2	C2S51_036102	XM_011080786.2
C2S51_017863	XM_011084006.2	C2S51_036246	XM_011084120.2
C2S51_018122	XM_011093646.2	C2S51_036246	XM_011081252.2
C2S51_018358	XM_011083277.2	C2S51_037514	XM_020698904.1
C2S51_019333	XM_011103049.2	C2S51_037725	XM_011084427.2
C2S51_019353	XM_011103188.2	C2S51_037958	XM_011084427.2
C2S51_019884	XM_011078850.2	C2S51_037958	XM_011101921.2
C2S51_019884	XM_020699262.1	C2S51_038307	XM_011103188.2

C2S51_020224	XM_011077550.2	C2S51_038307	XM_011076706.2
C2S51_020224	XM_011086288.2	C2S51_038314	XM_011103049.2
C2S51_020868	XM_020692310.1	C2S51_038468	XM_011076685.2
C2S51_020868	XM_011093336.2	C2S51_038468	XM_011085319.2
C2S51_022229	XM_011095049.2	C2S51_000426	XM_011095049.2

Table S6. Bacterial and yeast strains and plasmids used in this study.

Strain or plasmid	Description
Strains	
Escherichia coli DH5α	Strain used for plasmid construction
Agrobacterium GV3101	Overexpression of target genes mediated in tobacco
Y187 yeast strain	Strain used for Y1H assay
Plasmids	
pHIS2	Promoter bait vector for Y1H assay
pHIS2-P53	Positive control of bait vector for Y1H assay
pGADT7	Transcription factor prey vector for Y1H assay
pGAD53m	Positive control of prey vector for Y1H assay
pCAMBIA1300	Protein subcellular localization vector
pCAMBIA1301	Overexpression vector of promoter
pCAMBIA1303	Constituent plant overexpression vector
pJC-Gly-DSRB	Seed-specific plant overexpression vector

Table S7. Primer sequences used in this study.

Gene	Forward primer (5′→3′)	Reverse primer (5′→3′)
PfbZIP1-q	GGGGAAGGTGTTGATGGT	CCTCCAATGTCATCTCCC
PfbZIP4-q	CAGTCCTCGTCTGGCTTG	ATTTTCTTACCTCCCGCT
PfbZIP5-q	CGATCACCTCCTTAACCC	GAATCCCCGAACCATAGC
PfbZIP9-q	TCAATCTCCGCAGTTCCT	GCAACCCTCTTTCGTCTT
PfbZIP33-q	CAACACACACTTTGCGAG	GAGGCTCTGGGATGCTAA
PfbZIP48-q	AGCAATTCCGTGGATAGC	CGTTGCTTCAGTTTGGAG
PfbZIP71-q	TCTTCAGACAACTCGCCG	AGCCAAAGCCGCTATCC
PfbZIP91-q	AGTTGTCTGGCTCCGTAA	ACTTGTGGCACTTCTTCAT
PfbZIP85-q	CAGACTCCAGTTCACATACA	CTTCAGGCGGCTATTCTC
PfActin2-q	AGACCTTCAATGTGCCAGCCA	CACGACCAGCAAGATCCAACC
NtLPAT1-q	ACCTTGACCATTACTCCATT	GCATCGCTTGAGACATTC
NtDGAT2-q	GTTAGGACTTGCACCCGCTA	ACCAGTGGTTTGCCCATCTC
NtFAD2-q	AGCCGAAATCACAACTCGGA	GCCAAGCTAGCCCTTTTACCA
NtSAD1-q	ATGGCTGCTAGCCTTCGTTC	GATCCGCCCAGTCCTCCATA
NtFAD3-q	TCTGCACTGCCTTTGGTTCT	TTTGCTGCTTTGGTCGCTTC
NtLPCAT-q	CTCTGAAGCTGCTATCGTCGT	TCCTTCCTTTCTGTATCAGCCTC
NtDGAT1-q	GCACGCCAATTCATCAAG	AACATAGTAGTTCCGCAAGT
NtPDCT-q	CTTCTTCCCCGCCTTTCGAT	AAATCAACGCCTGACCCCAA
NtPDAT-q	ACCAGATGCACCTGACATGG	CGCTTAATGCAGGCACAGTC
NtDGAT3-q	GCAGAGGAGAAGGAGTTG	GTCCGAGGCTCAATCATAT
NtGPAT9-q	GGCTGTTGTCTGTGATGT	AACTCTGTTGCTTCCTCTC
NtLPAT2-q	CAGGTCTTCAACGGCTAA	TAGTGCGAGGAATCAATACA
NtActin-q	CAGTGGCCGTACAACAGGTA	AACCGAAGAATTGCATGAGG
GUS	TGGTGATGTGGAGTATTGC	CTGATGGTATCGGTGTGAG
bZIP52-	GTGGGCATCGATACGGGATCCA	ACGATTCATCTGCAGCTCGAGTCA
PGADT7	TGGATGGAGGTTGTGAGCAA	GTAAGGACAGCTGAAACTCCTC

bZIP85-PGADT7	GCCATGGAGGCCAGTGAATTCATGGCTAGAGCTACAGTAAATATTGGTG	CCGTATCGATGCCCACCCGGGTCATTCTTTAGGCCTTGCAAGC
roPfLPAT1B-1301	GACCTGCAGGCATGCAAGCTTAACAACCTTCAATAAAATAACCACAAG	TTACCCTCAGATCTACCATGGTGGTTACTATAATTTTGATTAATTTGCC
bZIP85-1300	ATACACCAAATCGACTCTAGAATGGCTAGAGCTACAGTAAATATTGGTG	GCCCTTGCTCACCATGGTACCTCATTCTTTAGGCCTTGCAAGC
bZIP85-1303	AACCTGCAGGTCGACTCTAGAATGGCTAGAGCTACAGTAAATATTGGTG	GGTTTAAACGAGCTCGGTACCTCATTCTTTAGGCCTTGCAAGC
bZIP85-DSRB	CACCATGTTGGGCCCGGCGCGCCATGGCTAGAGCTACAGTAAATATTGGTG	GTTGTCACATACATCGGCGCGCCTCATTCTTTAGGCCTTGCAAGC
